# Supplementary material for: ISOTOPE: ISOform-guided prediction of epiTOPEs in cancer
Source: PLoS Comput Biol. 2021 Sep 16;17(9):e1009411. doi: 10.1371/journal.pcbi.1009411 (PMC8478223; doi:10.1371/journal.pcbi.1009411)
Supplement: S1 Fig — Purity analysis of the small cell lung cancer (SCLC) samples from each one of the three cohorts used for this study: George et al. [24] (A), Iwakawa et al. [26] (B), and Rudin et al. [18] (C). In each case, we give the distribution of tumor purity values (between 0 and 1) calculated with ESTIMATE [41]. Length distributions of the new exons produced as a consequence of aberrant splice sites (D) or new exonizations (E). The lengths follow extreme value distributions with mean values of 100, similar to known exons. (PDF) [file pcbi.1009411.s001.pdf]

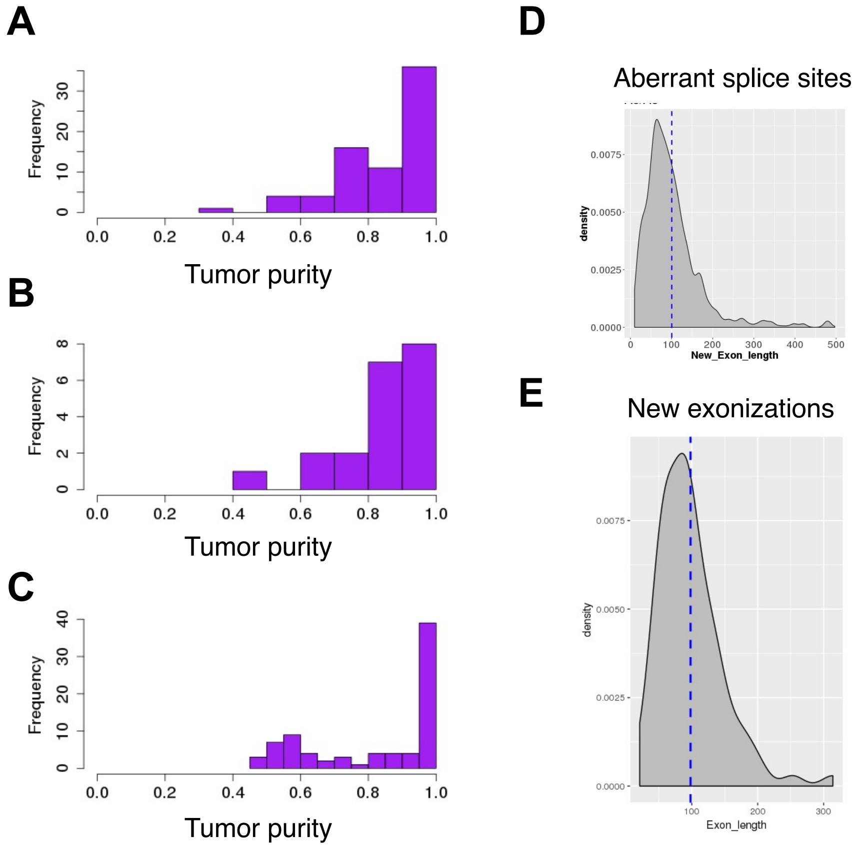

S1 Fig. Properties of the SCLC samples. Purity analysis of the small cell lung cancer (SCLC) samples from each one of the three cohorts used for this study: George et al. [1] (**A**), Iwakawa et al. [2] (**B**), and Rudin et al. [3] (**C**). In each case, we give the distribution of tumor purity values (between 0 and 1) calculated with ESTIMATE [4]. Length distributions of the new exons produced as a consequence of aberrant splice sites (**D**) or new exonizations (**E**). The lengths follow extreme value distributions with mean values of 100, similar to known exons.

References

1. George J, Lim JS, Jang SJ, Cun Y, Ozretić L, Kong G, et al. Comprehensive genomic profiles of small cell lung cancer. Nature. 2015;524: 47–53. doi:10.1038/nature14664
2. Iwakawa R, Kohno T, Totoki Y, Shibata T, Tsuchihara K, Mimaki S, et al. Expression and clinical significance of genes frequently mutated in small cell lung cancers defined by whole exome/RNA sequencing. Carcinogenesis. 2015;36: 616–621. doi:10.1093/carcin/bgv026
3. Rudin CM, Durinck S, Stawiski EW, Poirier JT, Modrusan Z, Shames DS, et al. Comprehensive genomic analysis identifies SOX2 as a frequently amplified gene in small-cell lung cancer. Nat Genet. 2012;44: 1111–1116. doi:10.1038/ng.2405
4. Yoshihara K, Shahmoradgoli M, Martínez E, Vegesna R, Kim H, Torres-Garcia W, et al. Inferring tumour purity and stromal and immune cell admixture from expression data. Nat Commun. 2013;4: 2612. doi:10.1038/ncomms3612
