# Supplementary material for: ISOTOPE: ISOform-guided prediction of epiTOPEs in cancer
Source: PLoS Comput Biol. 2021 Sep 16;17(9):e1009411. doi: 10.1371/journal.pcbi.1009411 (PMC8478223; doi:10.1371/journal.pcbi.1009411)
Supplement: S2 Fig — From top to bottom, the number of mapped spliced reads, the expression of the MYC genes (known to be amplified or overexpressed in SCLC and to drive splicing alterations), mutations on core spliceosome factors, tumor mutation burden and the number of the different event types detected by ISOTOPE for the SCLC samples (blue from George et al. [24], red from Rudin et al. [18], green from Iwakawa et al. [26]). (PDF) [file pcbi.1009411.s002.pdf]

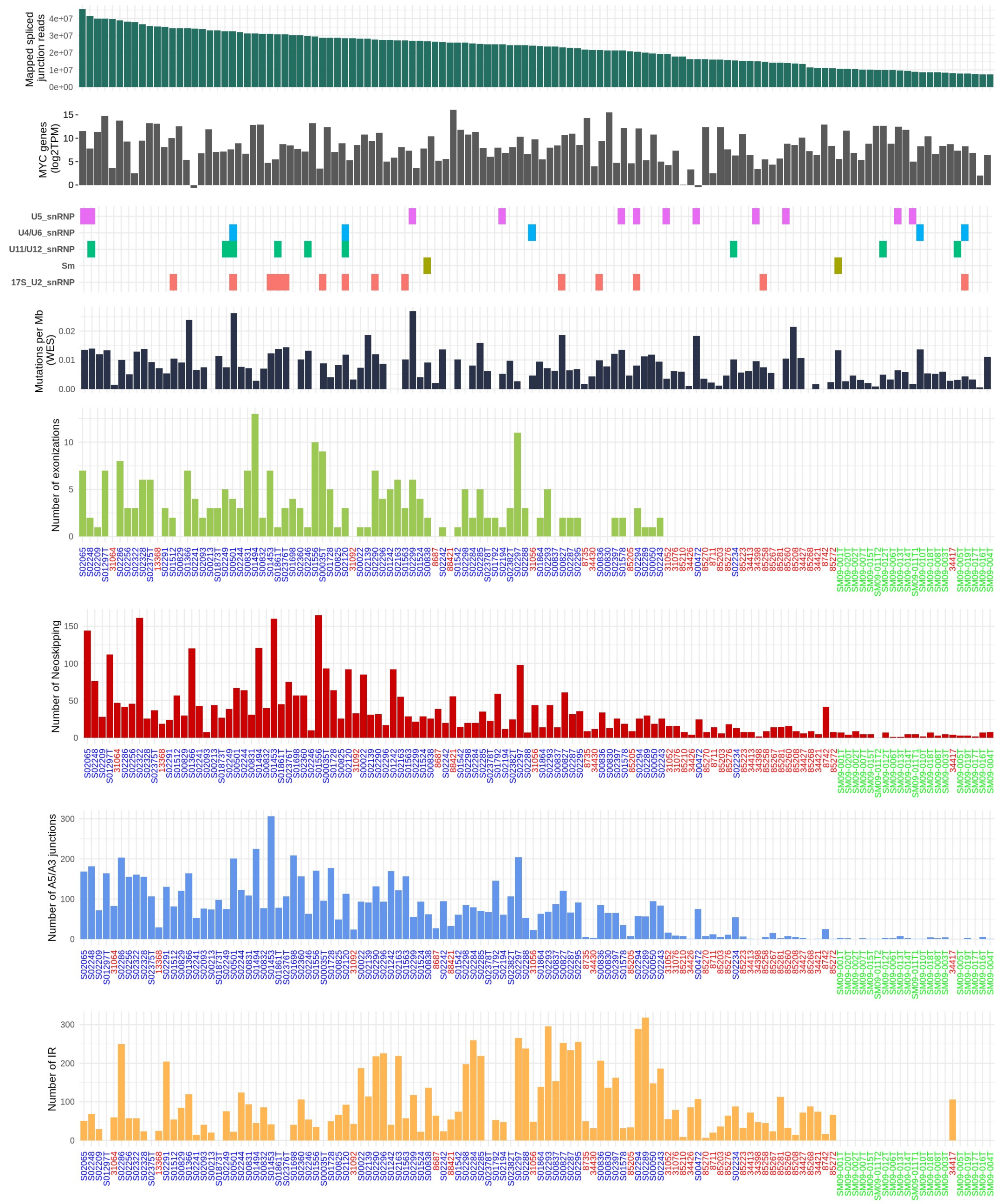

S2 Fig. SCLC-specific splicing alterations. From top to bottom, the number of mapped spliced reads, the expression of the MYC genes (known to be amplified or overexpressed in SCLC and to drive splicing alterations), mutations on core spliceosome factors, tumor mutation burden and the number of the different event types detected by ISOTOPE for the SCLC samples (blue from George et al. [1], red from Rudin et al. [2], green from Iwakawa et al. [3]).

References

1. George J, Lim JS, Jang SJ, Cun Y, Ozretić L, Kong G, et al. Comprehensive genomic profiles of small cell lung cancer. Nature. 2015;524: 47–53. doi:10.1038/nature14664
2. Rudin CM, Durinck S, Stawiski EW, Poirier JT, Modrusan Z, Shames DS, et al. Comprehensive genomic analysis identifies SOX2 as a frequently amplified gene in small-cell lung cancer. Nat Genet. 2012;44: 1111–1116. doi:10.1038/ng.2405
3. Iwakawa R, Kohno T, Totoki Y, Shibata T, Tsuchihara K, Mimaki S, et al. Expression and clinical significance of genes frequently mutated in small cell lung cancers defined by whole exome/RNA sequencing. Carcinogenesis. 2015;36: 616–621. doi:10.1093/carcin/bgv026
