# Supplementary material for: ISOTOPE: ISOform-guided prediction of epiTOPEs in cancer
Source: PLoS Comput Biol. 2021 Sep 16;17(9):e1009411. doi: 10.1371/journal.pcbi.1009411 (PMC8478223; doi:10.1371/journal.pcbi.1009411)
Supplement: S3 Fig — (A) Upper panel: Number of intron retentions per SCLC sample that impact the open reading frame. Lower panel: Number of candidate MHC-I binders per sample that are created (blue), i.e., splicing-derived neoepitopes, or potentially removed from the ORF by the splicing alteration (red) through exonizations. (B) Same as in (A) but for neoskipping events. (PDF) [file pcbi.1009411.s003.pdf]

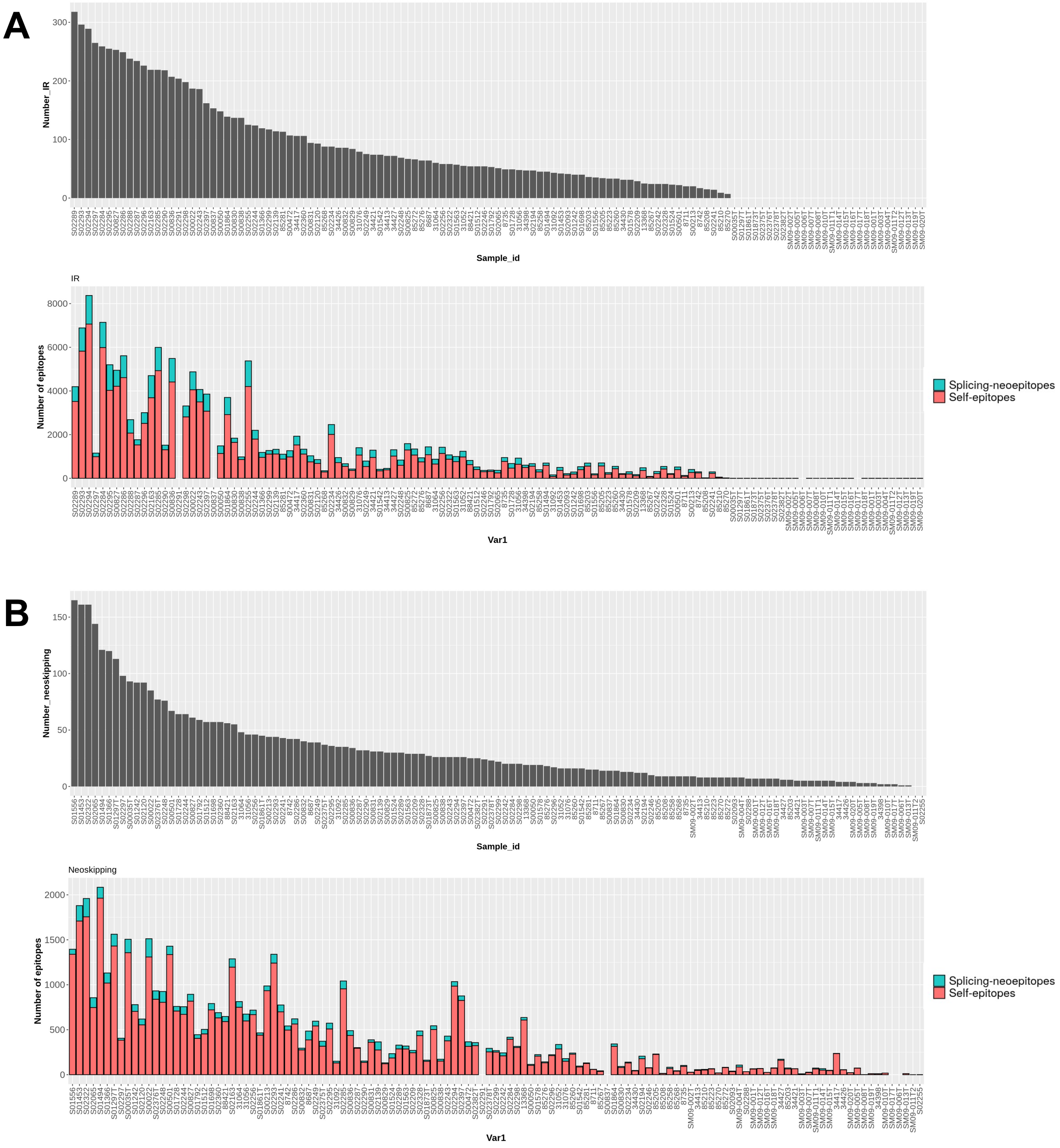

S3 Fig. Splicing-derived epitopes and splicing-affected self-epitopes in SCLC patients. (A) Upper panel: Number of intron retentions per SCLC sample that impact the open reading frame. Lower panel: Number of candidate MHC-I binders per sample that are created (blue), i.e., splicing-derived neoepitopes, or potentially removed from the ORF by the splicing alteration (red) through exonizations. **(B)** Same as in (A) but for neoskipping events.
