# Supplementary material for: ISOTOPE: ISOform-guided prediction of epiTOPEs in cancer
Source: PLoS Comput Biol. 2021 Sep 16;17(9):e1009411. doi: 10.1371/journal.pcbi.1009411 (PMC8478223; doi:10.1371/journal.pcbi.1009411)
Supplement: S4 Fig — (A) Correlations between the number of splicing alterations detected and the tumor mutation burden (TMB) for all the SCLC patients, separated by splicing alteration type. Although across all the events types the correlation is low (Spearman ρ = 0.182), separately there was a statistically significant correlation for Neoskipping events (ρ = 0.42). We show the same correlations separating splicing-derived neoepitopes (B) and splicing-affected self-epitopes. (C). Although neoskipping events showed significant association, there was an overall low correlation across all the event types between the TMB and the splicing-neoepitopes (ρ = 0.194) and self-epitopes (ρ = 0.196). (PDF) [file pcbi.1009411.s004.pdf]

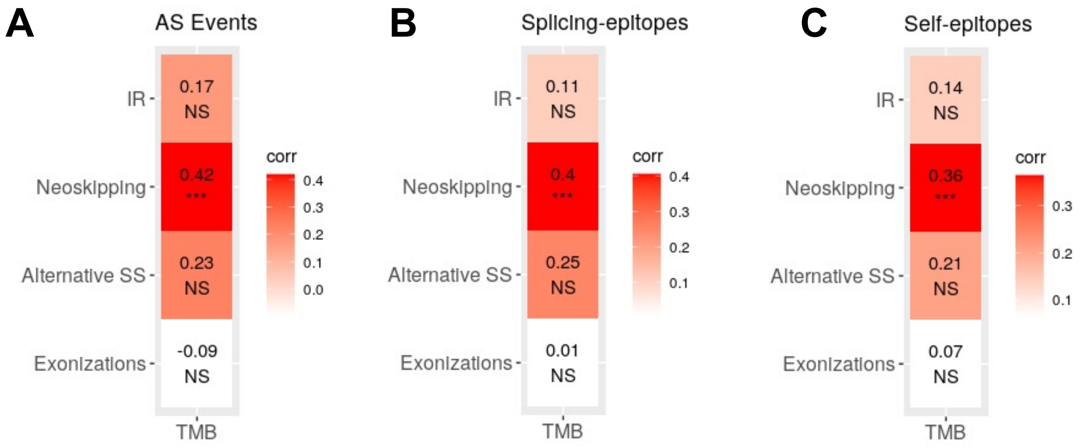

S4 Fig. Correlation of events and neoepitopes with the tumor mutation burden. (A) Correlations between the number of splicing alterations detected and the tumor mutation burden (TMB) for all the SCLC patients, separated by splicing alteration type. Although across all the events types the correlation is low (Spearman $\rho = 0.182$), separately there was a statistically significant correlation for Neoskipping events ($\rho = 0.42$). We show the same correlations separating splicing-derived neoepitopes **(B)** and splicing-affected self-epitopes. **(C).** Although neoskipping events showed significant association, there was an overall low correlation across all the event types between the TMB and the splicing-neoepitopes ($\rho = 0.194$) and self-epitopes ($\rho = 0.196$).
