# Supplementary material for: ISOTOPE: ISOform-guided prediction of epiTOPEs in cancer
Source: PLoS Comput Biol. 2021 Sep 16;17(9):e1009411. doi: 10.1371/journal.pcbi.1009411 (PMC8478223; doi:10.1371/journal.pcbi.1009411)
Supplement: S5 Fig — (A) Comparison of the number of neoepitopes derived from somatic mutations (red) and tumor-specific splicing-derived neoepitopes (blue) in the SCLC patient cohorts from Peifer et al. [25] and from George et al. [24] (B) For each patient from the same cohorts, we give the number of neoepitopes derived from somatic mutations (x axis) and the number of neoepitopes derived from tumor-specific splicing alterations (y axis). Mutation-derived epitopes were calculated with pVACtools, whereas splicing-derived neoepitopes were calculated with ISOTOPE as described in the manuscript. The candidate epitopes were calculated in both cases using the same tools with the same parameters: NetMHC and NetMHCPan, using the hg19 reference, testing peptides with amino acid length from 8 to 11, and selecting candidates with binding affinities less or equal than 500nM. There were no overlaps between candidates generated by both methods. (PDF) [file pcbi.1009411.s005.pdf]

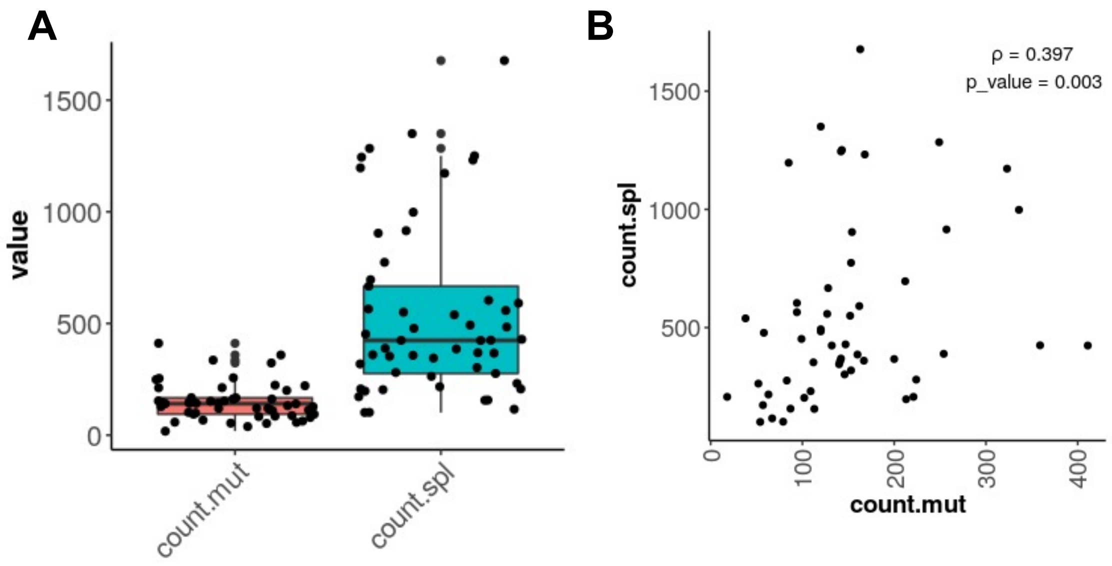

S5 Fig. Comparison between splicing-derived neoepitopes and neoepitopes derived from somatic mutations. (A) Comparison of the number of neoepitopes derived from somatic mutations (red) and tumor-specific splicing-derived neoepitopes (blue) in the SCLC patients from Peifer et al. [1] and George et al. [2] **(B)** For each patient from the same cohorts, we give the number of neoepitopes derived from somatic mutations (x axis) and the number of neoepitopes derived from tumor-specific splicing alterations (y axis). Mutation-derived epitopes were calculated with pVACtools, whereas splicing-derived neoepitopes were calculated with ISOTOPE as described in the manuscript. The candidate epitopes were calculated in both cases using the same tools with the same parameters: NetMHC and NetMHCPan, using the hg19 reference, testing peptides with amino acid length from 8 to 11, and selecting candidates with binding affinities less or equal than 500nM. There were no overlaps between candidates generated by both methods.

References

1. Peifer M, Fernández-Cuesta L, Sos ML, George J, Seidel D, Kasper LH, et al. Integrative genome analyses identify key somatic driver mutations of small-cell lung cancer. Nat Genet. 2012;44: 1104–1110. doi:10.1038/ng.2396
2. George J, Lim JS, Jang SJ, Cun Y, Ozretić L, Kong G, et al. Comprehensive genomic profiles of small cell lung cancer. Nature. 2015;524: 47–53. doi:10.1038/nature14664
