# Supplementary material for: ISOTOPE: ISOform-guided prediction of epiTOPEs in cancer
Source: PLoS Comput Biol. 2021 Sep 16;17(9):e1009411. doi: 10.1371/journal.pcbi.1009411 (PMC8478223; doi:10.1371/journal.pcbi.1009411)
Supplement: S6 Fig — We show the distributions of the length ratios between WT ORF and the ORF affected by the splicing alteration for the anti-PD1 (A) and the anti-CTLA4 (B) cohort. The ratios are plotted in log2 scale, i.e., log2(WT length/aberrant length). The plots are separated according to whether the change involved the creation of a splicing-derived neoepitope only (blue), the removal of a splicing-affected self-epitope only (green), or both (red). We plot in the lower panels the proportion of the total corresponding to each case. (PDF) [file pcbi.1009411.s006.pdf]

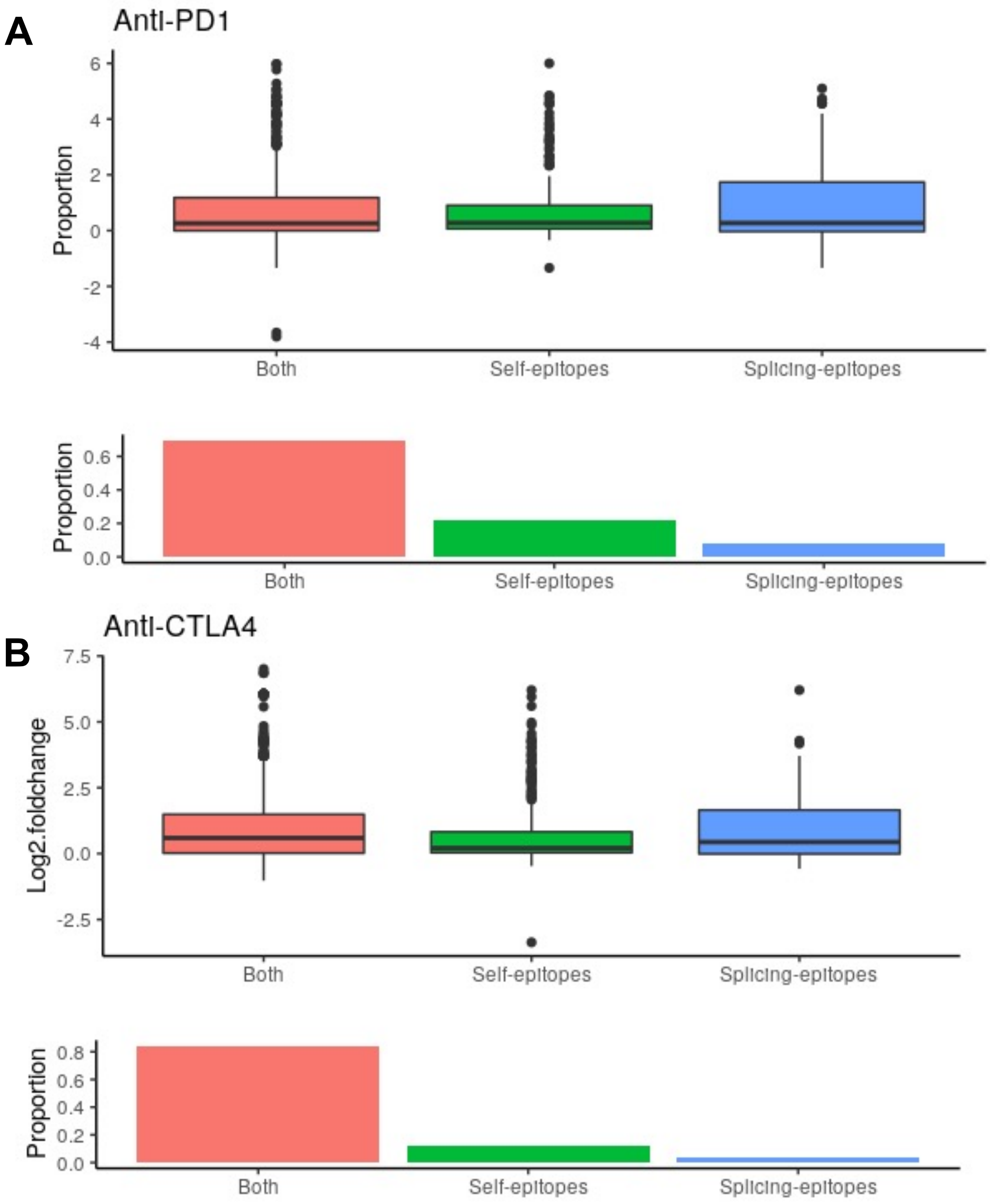

S6 Fig. Length differences between the wild type (WT) open reading frame (ORF) and the splicing altered ORF. We show the distributions of the length ratios between WT ORF and the ORF affected by the splicing alteration for the anti-PD1 **(A)** and the anti-CTLA4 **(B)** cohort. The ratios are plotted in log2 scale, i.e., log2(WT length / aberrant length). The plots are separated according to whether the change involved the creation of a splicing-derived neoepitope only (blue), the removal of a splicing-affected self-epitope only (green), or both (red). We plot in the lower panels the proportion of the total corresponding to each case.
