# Supplementary material for: ISOTOPE: ISOform-guided prediction of epiTOPEs in cancer
Source: PLoS Comput Biol. 2021 Sep 16;17(9):e1009411. doi: 10.1371/journal.pcbi.1009411 (PMC8478223; doi:10.1371/journal.pcbi.1009411)
Supplement: S7 Fig — (A) Distribution of the number of candidate tumor-specific splicing-derived neoepitopes (splicing-epitopes) and splicing-affected self-epitopes that would be depleted in the altered isoform (self-epitopes) using ≤300nM to define candidate epitopes. (B) Distribution of the number of candidate epitopes from (A), separated by HLA-type. (PDF) [file pcbi.1009411.s007.pdf]

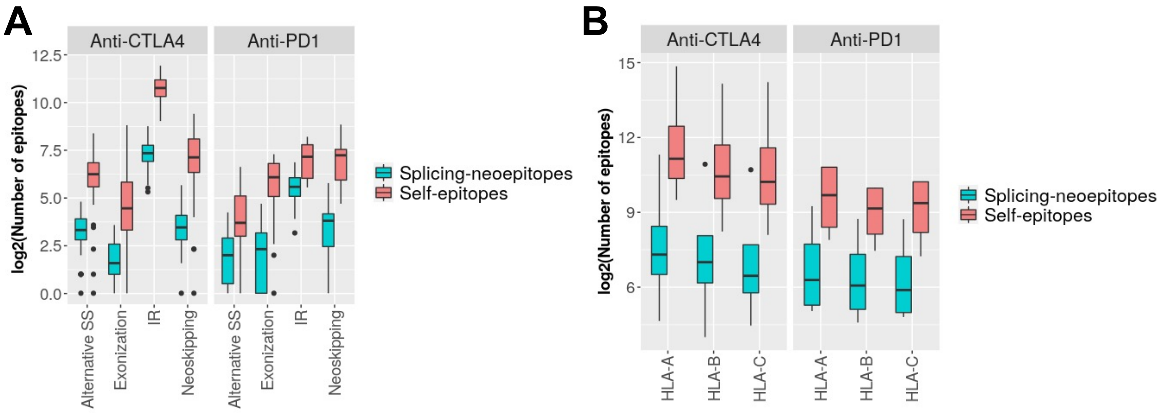

S7 Fig. Splicing-associated epitopes identified using ≤300nM. **(A)** Distribution of the number of candidate tumor-specific splicing-derived neoepitopes (splicing-epitopes) and splicing-affected self-epitopes that would be depleted in the altered isoform (self-epitopes) using ≤300nM to define candidate epitopes. **(B)** Distribution of the number of candidate epitopes from (A), separated by HLA-type.
