# Supplementary material for: ISOTOPE: ISOform-guided prediction of epiTOPEs in cancer
Source: PLoS Comput Biol. 2021 Sep 16;17(9):e1009411. doi: 10.1371/journal.pcbi.1009411 (PMC8478223; doi:10.1371/journal.pcbi.1009411)
Supplement: S8 Fig — (A) Distribution of the number of tumor-specific splicing-derived neoepitopes (splicing-epitopes) and splicing-affected self-epitopes that would be depleted in the altered isoform (self-epitopes) using ≤500nM to define candidate epitopes, separated by clinical outcome. The number of splicing-derived neoepitopes in responders to anti-CTLA4 (mean 346) and non-responders (mean 375) were not significantly different. Similarly, the anti-PD1 cohort showed no significant difference between the total number of splicing-derived neoepitopes between responders (mean 46) and non-responders (mean 62.6) in the anti-PD1 cohort. (B) as in (A) but using ≤300nM to define candidates. The number of tumor-specific splicing-derived epitopes in responders to anti-CTLA4 (median 172) and non-responders (median 228) were not significantly different. A similar result was found for the self-epitopes (2090 and 2159). We found the same for the anti-PD1 cohort (splicing tumor-epitopes: 51.5 vs 79; splicing self-epitopes: 269 vs 287). (C) Proportion of splicing-affected self-epitopes over the total of epitopes (splicing-affected self-epitopes and tumor-specific splicing-derived neoepitopes) (y axis) for patients treated with anti-CTLA4, separated by type of splicing alteration (x axis) and by patient response: responder (green) or non-responder (red). In this plot, candidate epitopes were defined using ≤300nM as threshold. (D) As in (B) but for melanoma patients treated with anti-PD1. (PDF) [file pcbi.1009411.s008.pdf]

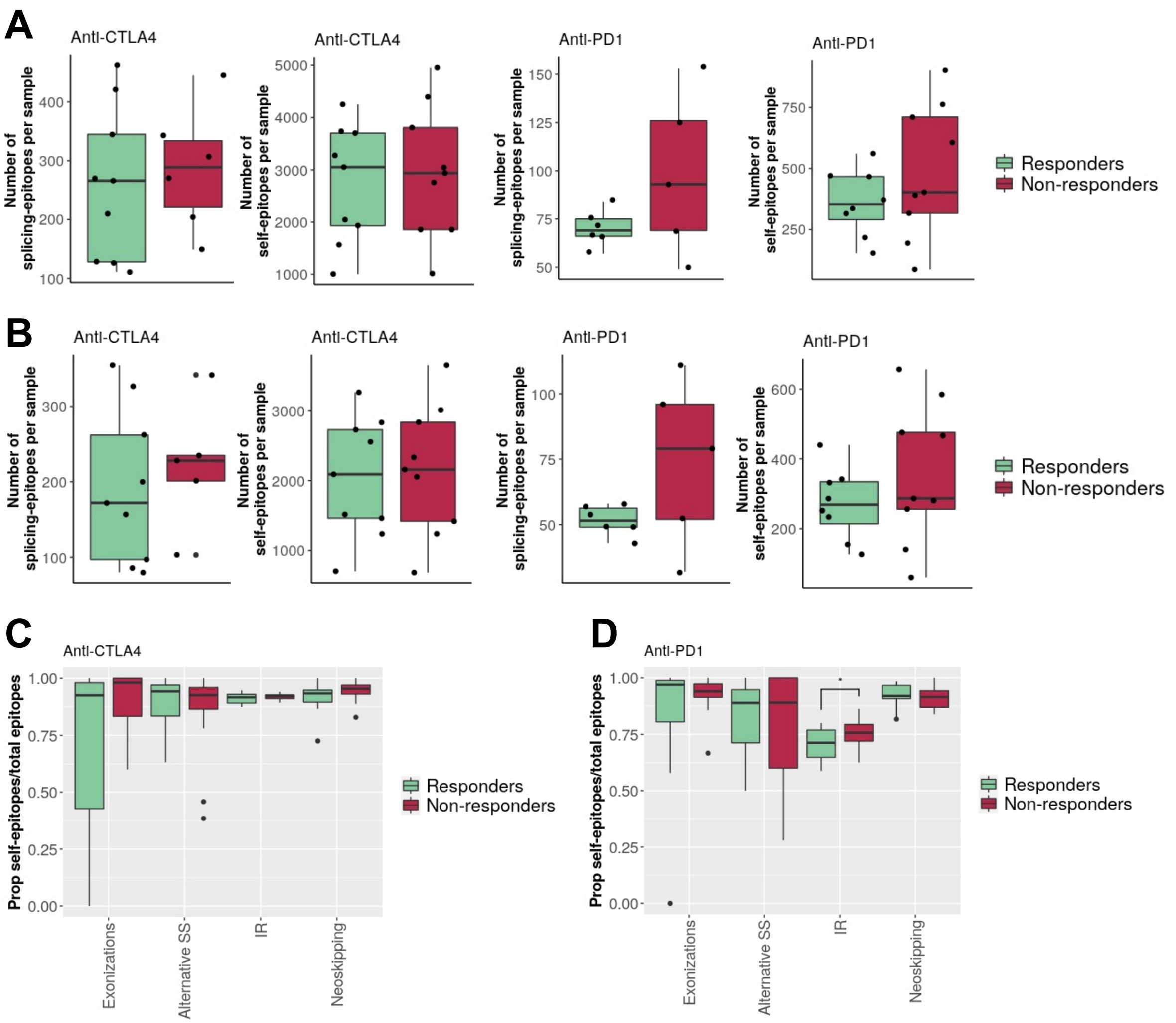

S8 Fig. Splicing-associated epitopes and immune therapy response. (A) Distribution of the number of tumor-specific splicing-derived neoepitopes (splicing-epitopes) and splicing-affected self-epitopes that would be depleted in the altered isoform (self-epitopes) using ≤500nM to define candidate epitopes, separated by clinical outcome. The number of splicing-derived neoepitopes in responders to anti-CTLA4 (mean 346) and non-responders (mean 375) were not significantly different. Similarly, the anti-PD1 cohort showed no significant difference between the total number of splicing-derived neoepitopes between responders (mean 46) and non-responders (mean 62.6) in the anti-PD1 cohort. **(B)** as in (A) but using ≤300nM to define candidates. The number of tumor-specific splicing-derived epitopes in responders to anti-CTLA4 (median 172) and non-responders (median 228) were not significantly different. A similar result was found for the self-epitopes (2090 and 2159). We found the same for the anti-PD1 cohort (splicing tumor-epitopes: 51.5 vs 79; splicing self-epitopes: 269 vs 287). **(C)** Proportion of splicing-affected self-epitopes over the total of epitopes (splicing-affected self-epitopes and tumor-specific splicing-derived neoepitopes) (y axis) for patients treated with anti-*CTLA4*, separated by type of splicing alteration (x axis) and by patient response: responder (green) or non-responder (red). In this plot, candidate epitopes were defined using ≤300nM as threshold. **(D)** As in (B) but for melanoma patients treated with anti-*PD1*.
