# Supplementary material for: ISOTOPE: ISOform-guided prediction of epiTOPEs in cancer
Source: PLoS Comput Biol. 2021 Sep 16;17(9):e1009411. doi: 10.1371/journal.pcbi.1009411 (PMC8478223; doi:10.1371/journal.pcbi.1009411)
Supplement: S9 Fig — (A) Cumulative plot of the binding affinities (x axis) of splicing-affected self-epitopes in melanoma tumors from exonization events separated in responders (green) and non-responders (red) to anti-PD1 therapy. Smaller values of binding affinity correspond to a stronger interaction between the peptides and the MHC-I complex. We also give the Kolmogorov-Smirnov test p-value (KS) = 0.0074. (B) Cumulative plot of the binding affinities (x axis) of splicing-affected self-epitopes in melanoma tumors from new skipping events (neoskipping) events separated in responders (green) and non-responders (red) to anti-CTLA4 therapy, KS = 0. (C) Cumulative plots of the affinities of splicing-affected self-epitopes in melanoma tumors from intron retention events separated in responders (green) and non-responders (red) to anti-CTLA4 therapy, KS = 0. (PDF) [file pcbi.1009411.s009.pdf]

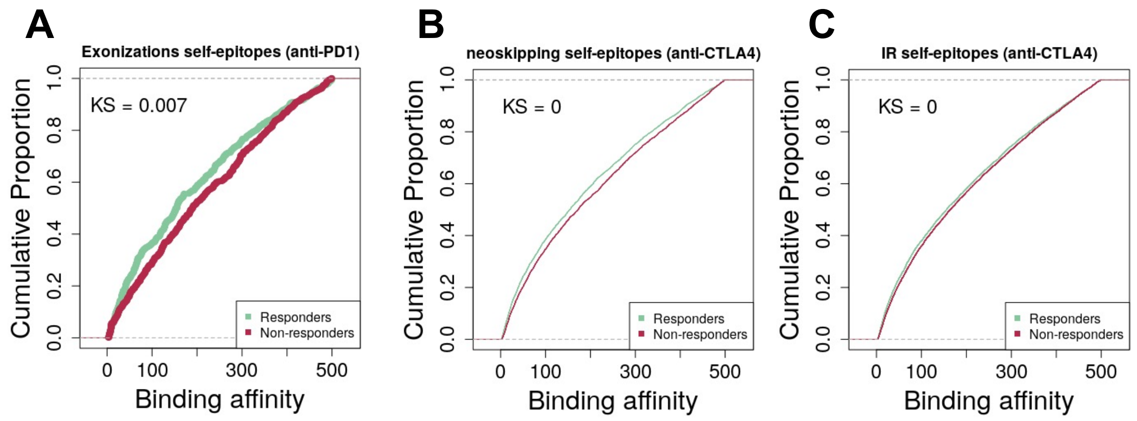

S9 Fig. Analysis of the epitope affinities in responders and non-responders using ≤500nM to define candidates. (A) Cumulative plot of the binding affinities (x axis) of splicing-affected self-epitopes in melanoma tumors from exonization events separated in responders (green) and non-responders (red) to anti-PD1 therapy. Smaller values of binding affinity correspond to a stronger interaction between the peptides and the MHC-I complex. We also give the Kolmogorov-Smirnov test p-value (KS) = 0.0074. **(B)** Cumulative plot of the binding affinities (x axis) of splicing-affected self-epitopes in melanoma tumors from new skipping events (neoskipping) events separated in responders (green) and non-responders (red) to anti-*CTLA4* therapy, KS = 0. **(C)** Cumulative plots of the affinities of splicing-affected self-epitopes in melanoma tumors from intron retention events separated in responders (green) and non-responders (red) to anti-*CTLA4* therapy, KS = 0.
