# Supplementary material for: ISOTOPE: ISOform-guided prediction of epiTOPEs in cancer
Source: PLoS Comput Biol. 2021 Sep 16;17(9):e1009411. doi: 10.1371/journal.pcbi.1009411 (PMC8478223; doi:10.1371/journal.pcbi.1009411)
Supplement: S11 Fig — We show the number of mapped reads in responder and non-responder patients in the anti-CTLA4 (A) and the anti-PD1 (B) cohorts. We also show the distribution of the transcript expression values for each patient, represented as log2(TPM) (y axis) for the anti-CTLA4 (C) and for the anti-PD1 (D) cohorts. (PDF) [file pcbi.1009411.s011.pdf]

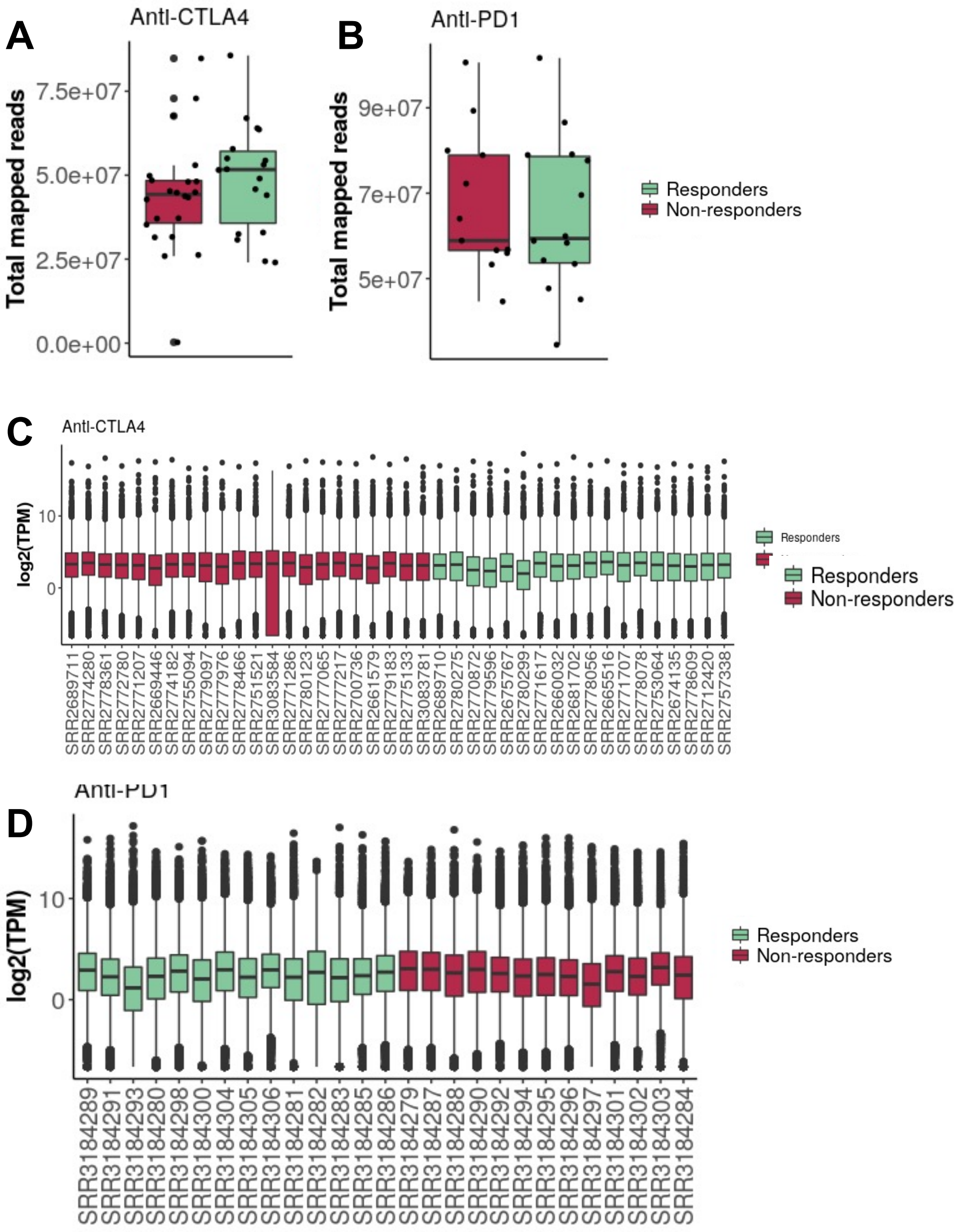

S11 Fig. Comparison of sample properties between responders and non-responders. We show the number of mapped reads in responder and non-responder patients in the anti-CTLA4 **(A)** and the anti-PD1 **(B)** cohorts. We also show the distribution of the transcript expression values for each patient, represented as log2(TPM) (y axis) for the anti-*CTLA4* **(C)** and for the anti-*PD1* **(D)** cohorts.
