# Supplementary material for: ISOTOPE: ISOform-guided prediction of epiTOPEs in cancer
Source: PLoS Comput Biol. 2021 Sep 16;17(9):e1009411. doi: 10.1371/journal.pcbi.1009411 (PMC8478223; doi:10.1371/journal.pcbi.1009411)
Supplement: S12 Fig — We show the stromal content (StromalScore), immune cell infiltration (ImmuneScore), and overall score predicted with ESTIMATE separating patients according to the treatment response in each cohort, anti-PD1 (A) and anti-CTLA4 (B). The only significant differences detected was in relation to the stromal content in the anti-PD1 cohort (p-value ~ 0.05). (C) Number of splicing neo-epitopes (y axis) as a function of the stromal score (x axis). (PDF) [file pcbi.1009411.s012.pdf]

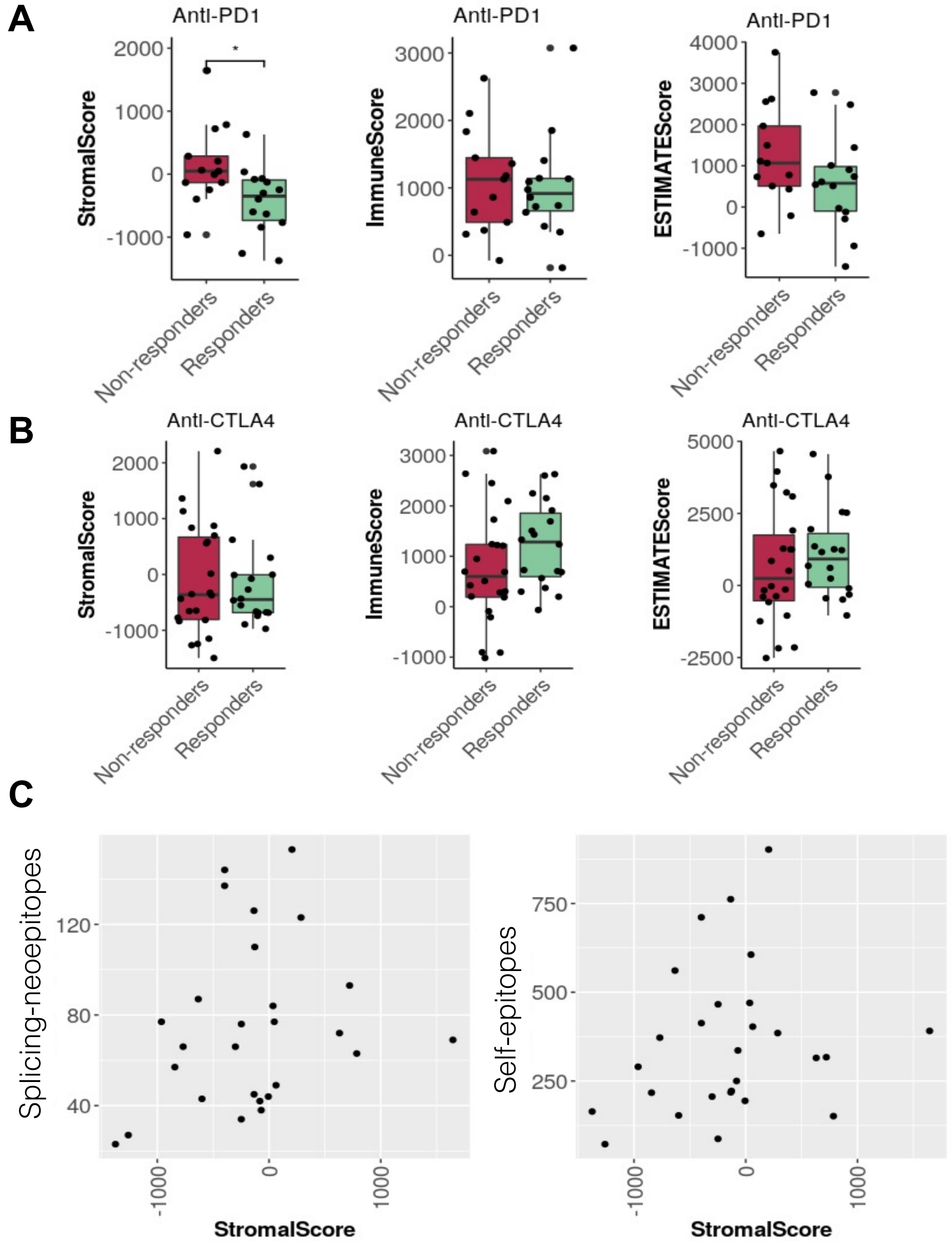

S12 Fig. Stroma and Immune content comparisons between responders and non-responders. We show the stromal content (StromalScore), immune cell infiltration (ImmuneScore), and overall score predicted with ESTIMATE separating patients according to the treatment response in each cohort, anti-*PD1* **(A)** and anti-*CTLA4* **(B)**. The only significant differences detected was in relation to the stromal content in the anti-PD1 cohort (p-value ~ 0.05). **(C)** Number of splicing neo-epitopes (y axis) as a function of the stromal score (x axis).
