# Supplementary material for: ISOTOPE: ISOform-guided prediction of epiTOPEs in cancer
Source: PLoS Comput Biol. 2021 Sep 16;17(9):e1009411. doi: 10.1371/journal.pcbi.1009411 (PMC8478223; doi:10.1371/journal.pcbi.1009411)
Supplement: Attachment — Submitted filename: answer_to_reviewers_v22.pdf [file pcbi.1009411.s020.pdf]

Reviews

We cannot make any decision about publication until we have seen the revised manuscript and your response to the reviewers' comments. Your revised manuscript is also likely to be sent to reviewers for further evaluation.

In melanoma it is known that in some cases immune response is targeted against self-antigens in genes involved in pigmentations, for example PMEL (SILV), MLANA, and such immune response is associated with loss, of variable degree, of skin pigmentation. There is also some association between degree of autoimmune side effects and therapeutic benefits from immune check point inhibitors. However, as indicated by reviewers there are number of questions which need to be carefully addressed regarding possibility of loss of self-antigens due to splicing and putting such potential mechanism in context of other known mechanisms of immune escape.

ISOTOPE method as indicated by reviewers has potential to generate useful analysis, however there are number of questions raised by reviewers which need to be carefully addressed. One of areas which needs to be addressed is providing much more details on method, including important relevant aspects of existing tools used by this method, for example exon junction reads in context of exon skipping analysis. Also, including information potential method users likely would want to be included, for example information on transcripts MANE Select/Plus status, gene names, etc.

We have tried to improve the description of the method and analyses performed. We have completed the details about the methodologies in the Method section. Moreover, in ISOTOPE's GitHub page (https://github.com/comprna/ISOTOPE) we have described in detail the pipeline with the specific commands to be used step by step, and have provided an example dataset for users to test the pipeline.

We have clarified the scope of the results and have toned down the conclusions in relation to the evidence for an impact by the loss of self-epitopes. Accordingly, we now described them as splicing-neoepitopes and splicing-affected self-epitopes/ We have extended the results with new analyses, including the comparison with mutational-neoepitopes.

The added details in this revised version include the existing and novel programs used to analyse the spliced junctions, the test for significance of the spliced junctions, the analysis of how they affect the protein encoded by the host gene, and the extraction of candidate epitopes. The pipeline uses the annotation from Ensembl, and the outputs provide the Ensembl gene IDs as reference. The use of Ensembl provides several advantages over other annotation sets. For instance, MANE is a minimal set of matching RefSeq and Ensembl transcripts, so it is a subset of the Ensembl annotation. They would be already included in our annotation set but would not provide a complete repertoire of possible normal transcript isoforms. On the other hand, the advantage of Ensembl over RefSeq is that Ensembl, unlike RefSeq, provides an annotation of gene loci with unique per-locus identifiers, which is more useful to extract information of the potential generation of epitopes from the processing of RNAs expressed from the genome and to relate to external datasets, like the database of genes associated to therapy response used in our manuscript.

Reviewer's Responses to Questions

Comments to the Authors:

Please note here if the review is uploaded as an attachment.

Reviewer #1: Manuscript Review: ISOTOPE: ISOform-guided prediction of epiTOPES in cancer

Summary

The authors present a method, “ISOTOPE” for identifying splice isoform variants in RNA-seq samples derived from cancer patients. The authors present data on the validation of this method, and then apply it to large genomic data sets to identify patterns of different splice isoforms in cancer. A recurrent theme is how splice isoforms lead to depletion of “self antigens”, which the authors reference numerous times.

In general, while the method itself is potentially interesting, the analysis of results generated using this method is problematic with a range of issues and questions detailed below. Claims around the depletion of self antigens are confusing and it is unclear if there is a particular significance of this and also, what the appropriate comparison group is. I have included my notes below - as it stands there are so many issues with this manuscript I have only listed the most pressing ones here.

Thank you for raising these questions about the manuscript. We have now toned down these claims about the significance of the depleted self-antigens, as the evidence for their role in tumor immunity is not robust enough. We have changed the labels of the types of epitopes to reflect that. The manuscript now describes *tumor-specific splicing-derived neoepitopes*, or *splicing-neoepitopes* for short, to refer to candidate epitopes that are potentially created by the splicing alteration; and *splicing-affected self-epitopes*, or *self-epitopes* for short, to refer to native epitopes that would not be encoded by the altered isoform in the tumor.

We have also provided additional information about the methods implemented, as well as a tutorial and sample datasets to test the code in ISOTOPE’s GitHub page https://github.com/comprna/ISOTOPE

Concerns:

Intro: Does this method require a special/certain type of sequencing? How can it identify *all* cancer-specific splicing alterations? That is a strong claim.

ISOTOPE works with standard Illumina paired-end sequencing. We have used samples ranging between 20M and 120M reads per sample. Regarding read-length, the analyzed samples were 100nt except for the samples from Iwakawa et al, which were 50nt (paired end).

The advantage of ISOTOPE is that it can evaluate one individual tumor sample. By saying that “*we identify all cancer-specific splicing alterations*” we meant that we can calculate all the splicing junctions that occur in a given tumor sample that are different from those observed in a comprehensive set of normal samples from multiple tissues and individuales. These comprehensive set of normal junctions were calculated from the mapped RNA-seq data from GTEX (v2.0, nearly 10,000 RNA-seq samples), the CHESS annotations, the junctions from normal samples in Intropolis, and the junctions from 24

matched normal samples from lung tissues from the Rudin et al. 2012 study. We have improved the description of the normal datasets in the Results and Methods section to clarify this point.

The description of the method in the main text is highly perfunctory and confusing. Would suggest including more detail here and also referencing the methods more heavily.

We have tried to improve the description of the method and analyses performed. We have completed the details about the methodologies in the Method section, where we reference all methods used. Moreover, in ISOTOPE's GitHub page (https://github.com/comprna/ISOTOPE) we have described in detail the pipeline with the specific commands to be used step by step, and have provided an example dataset for users to test the pipeline.

Method validation: it would seem to be important for the authors' approach that they can in fact identify bonafide neoepitopes with their method. They claim in their validation section that "We were able to detect three neoepitopes on three different samples" - this feels rather weak to me, given that 100s or 1000s of neoepitopes are being predicted. For any method, I would expect some to hit even by chance. The authors should do a more thorough analysis demonstrating that their approach can indeed predict and prioritize true tumor presented epitopes better than previous and/or naive approaches.

There are multiple limitations in the mass-spectrometry for immunopeptidomics, such as the required starting amount of material and the difficulty to establish the specificity of the observed peptides. Consequently, the validation rate is low in all studies published so far. Additionally, ours is not a usual identification of epitopes from immunopeptidomics as it is only based on the new peptides produced by splicing patterns that are exclusively found in the tumor samples. Accordingly, the rate of validation is expected to be low.

Our level of validation is similar to previous methods based on MHC-associated mass-spec: e.g. Smart et al. 2018 only validated 3 peptides from immunopeptidomics from three cell lines analysed. Kahles et al 2018 found more candidates in a large set of patients, but they used standard mass-spectrometry data. Whether these peptides were associated with the MHC-I complex remains to be determined.

When we compared self-epitopes with MHC-associated mass-spec from B-cells (in section "Tumor-specific splicing alterations frequently affect self-epitopes"), the number of validated epitope predictions was much higher: 1458 (11.7% of the predicted epitopes). These were peptides generated from annotated transcripts generally expressed in normal samples, predicted not to be present in the altered transcript isoform in the tumor, and estimated to have affinity to the MHC-I class complex for the HLA type of the same sample.

Ours and previous analyses suggest that there is a small contribution of tumor-specific splicing to novel epitopes, and that the majority of peptides associated to the immune recognition of tumor samples may be produced through other mechanisms or may not be "novel" from the point of view of the expression pattern. We have mentioned this possibility in the discussion section of the revised manuscript.

“Gained and depleted enrichment analysis”: the claim there are genes “enriched” for depleted self-antigens (ATM, EZH2, REL1) is a very strong one, since this would imply recurrent immune selection against a presumably wildtype peptide. If this is true substantially more evidence, included as figures and statistical analyses, is required. In general, the presentation of anecdotal evidence for a subset of genes like above is not sufficient for a claim, since it is unknown how many total genes were tested in this setting.

Apologies if this text was confusing. We have rewritten this part to try to make it more clear. We did not mean to say that these genes were enriched for “depleted self-antigens”. This text describes the association of genes linked to therapy response with the patterns observed for epitopes. We found that there is a significant overlap between the set of genes associated with therapy response in lymphoma and the set of genes with depleted self-epitopes compared with the genes with gained epitopes. The possible clinical implications of this overlap are out of the scope of this manuscript.

Neoantigen prediction:

“Peptides in the altered protein with binding affinity ≤ 500nM, but not present in the reference proteome were considered candidate neo-epitopes.”: 500 nM is a very weak cutoff and much stronger cutoffs have been recently proposed [cell]. Please show your analysis and conclusions are not dependent on this value.

We agree with the reviewer that 500nM might not be stringent enough. Most of our predictions lie between 100nM and 500nM. The threshold 500nM has been used in several recent articles, e.g. https://pubmed.ncbi.nlm.nih.gov/30894752/, https://pubmed.ncbi.nlm.nih.gov/29107330/ and it was also the cutoff used for candidates in the NetHMC paper (https://pubmed.ncbi.nlm.nih.gov/26515819/).

To address the reviewer’s question, we have reassessed our findings lowering the threshold to 300nm. The results remain the same.

Using 300nM, we still observed a frequency of self-epitopes that are potentially depleted, compared with the number of tumor-specific splicing-derived epitopes:

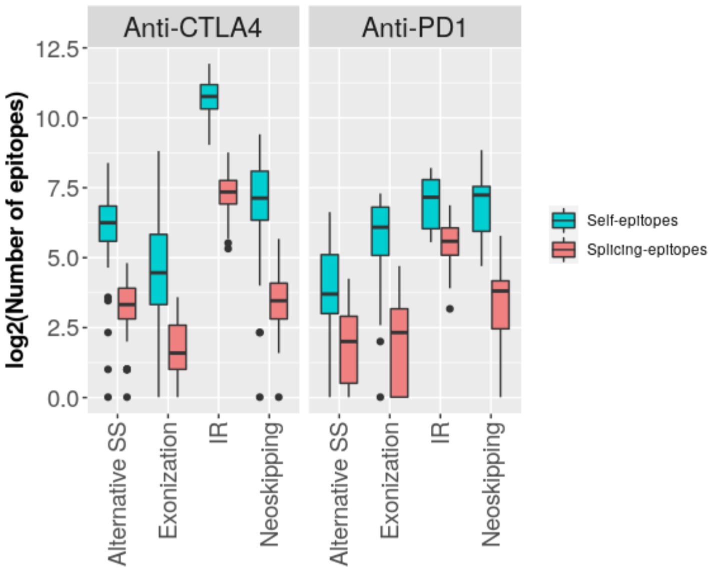

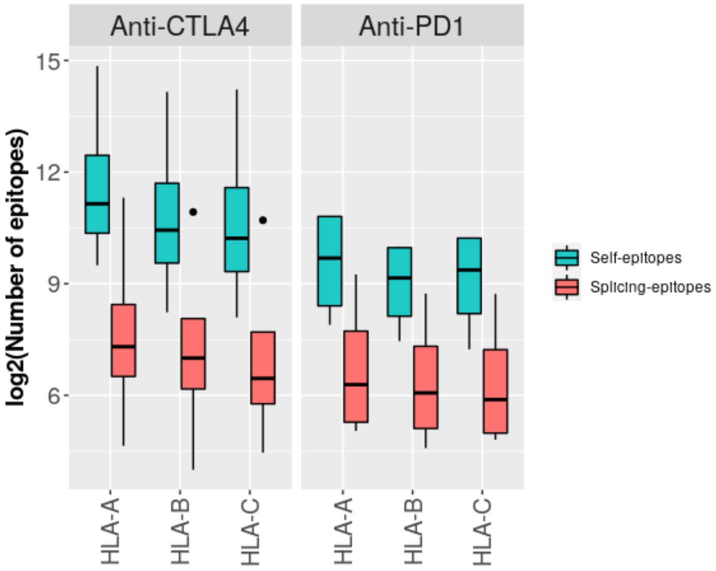

We have added this information in the Supp. Figure 7.

The number of tumor-specific splicing-derived epitopes in responders and non-responders in both cohorts were not significantly different. We plot below these distributions:

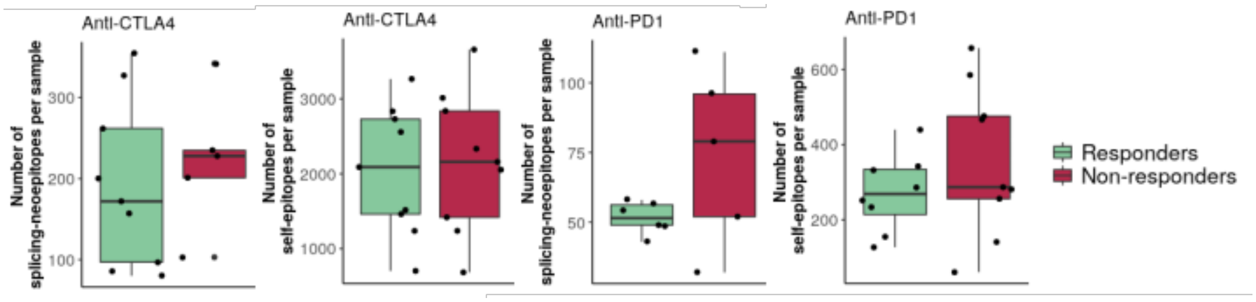

These plots for the 300nM and the 500nM are now included in Supp. Fig. 8.

Additionally, the proportion of self-epitopes that are affected by the splicing alteration from the total is similar as before in responders and non-responders, and we also recovered the same significant difference in the case of neoepitopes produced through IR events (p = 0.05):

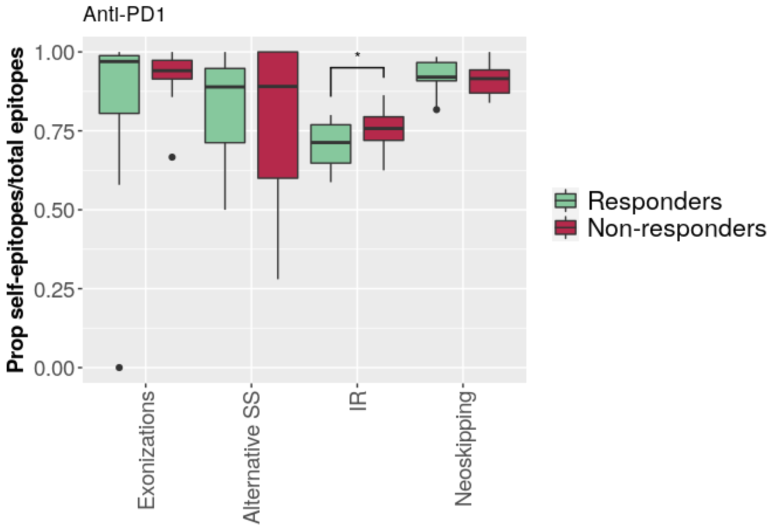

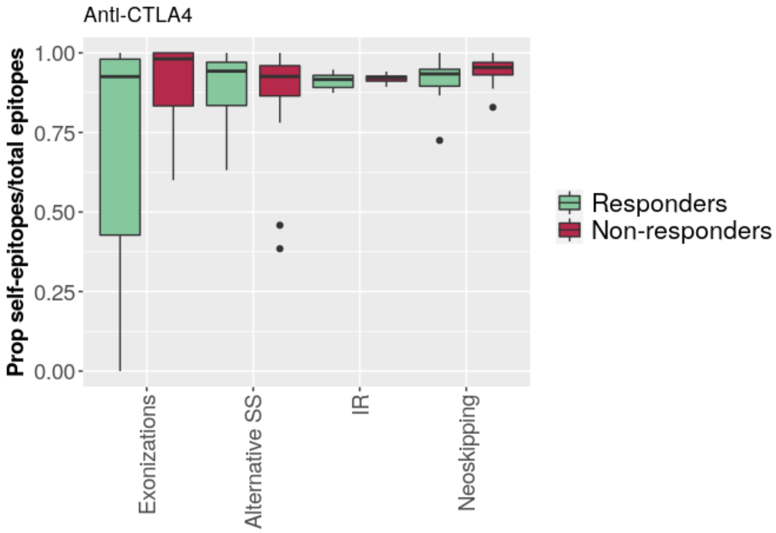

We have included these images as Supp. Fig. 8, and now the text mentions these findings using 300nM as threshold.

Regarding the differences observed in the binding affinities, some of the comparisons are not significant anymore, but we still observe similar trends:

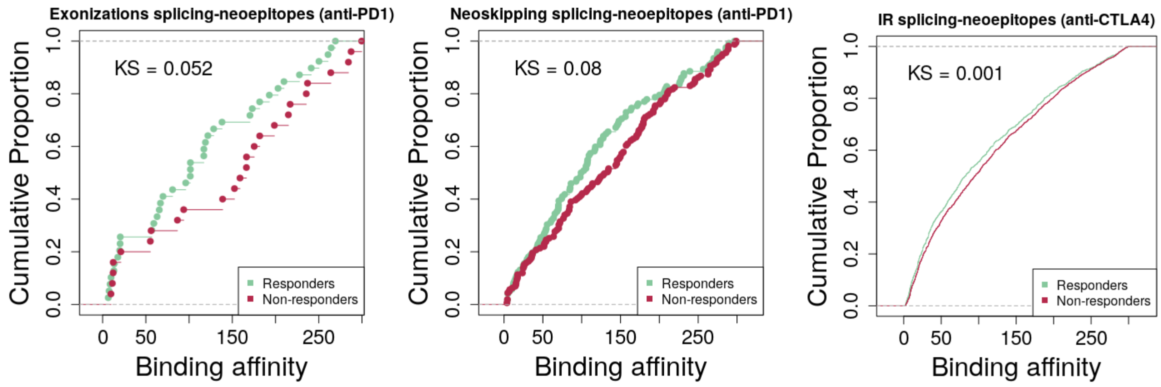

We have added these plots as Supp. Fig. 10.

The significant differences observed for epitopes originating from IR events could be due to the high abundance of the IR events. The differences observed in binding affinity are mainly caused by epitopes with affinities between 100nM and 500nM.

Furthermore, expression level is known to be a key determinant of tumor epitope immunogenicity. Since your calls are made from RNA-seq, I would strongly recommend including an expression estimate for your candidate splice-isoform derived neoepitopes. Notably, since these can co-exist with the wildtype isoform, it will be important to establish how the expression of these epitopes can be assessed in an isoform-specific manner.

For each splicing event not in the normal samples, we identified the most highly expressed normal transcript in the corresponding gene. This is the transcript used as reference to check if the event would change the open reading frame. We considered that including splicing events from regions with low abundance may produce splicing changes whose functional impact might be uncertain. Accordingly, the reference transcript is generally still expressed in the tumor sample. However, we are performing the analysis at the level of the relative abundance. That is, we measured whether the tumor-specific splicing has a relative abundance that is significant with respect to the expression of the locus.

The significance for each tumor-specific splicing event found was assessed in the following way: we sampled random regions of the same size as the event across the gene locus in which the event was located. We then compared the read coverage of our event with the empirical distribution of coverages obtained from the random regions to test the significance. This test is explained in detail in the tutorial provided in the ISOTOPE's GitHub page https://github.com/comprna/ISOTOPE

We have looked at the distribution of the expression (in TPM units) of the reference isoforms comparing patients with and without a significant tumor-specific splicing change. That is, for each gene with a significant event, we compared the expression distribution for patients with and without significant events using a t-test if the distributions were normal and a wilcoxon test otherwise.

Between 7 and 10% of the tested genes showed that the reference transcript had significantly lower expression in patients with the tumor-specific event:

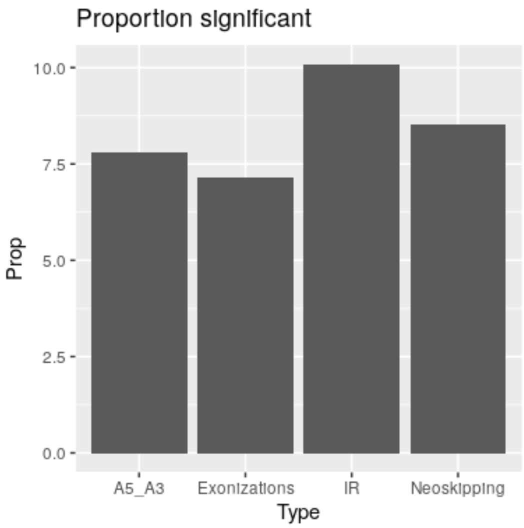

On the other hand, between 10 and 25% of the tested genes showed that the reference transcript had significantly higher expression in patients with the tumor-specific event:

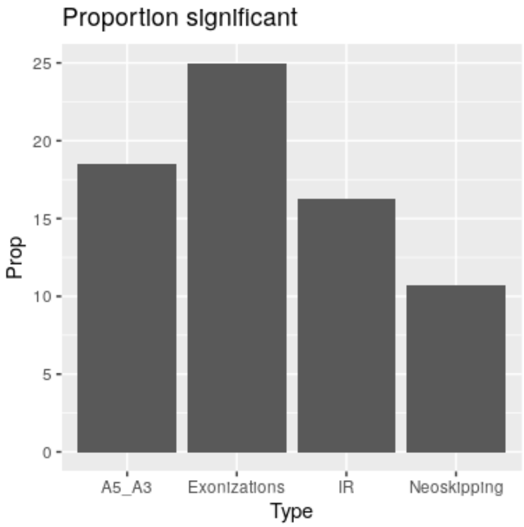

Thus in the majority of cases the transcript does not change expression, and only a low proportion (7-10%) decrease expression, in terms of the abundance. However, in terms of the relative abundance, our tests indicate that there is an increase of the tumor-specific junction. This can still happen regardless of the global abundance of the normal transcripts. Nonetheless, we agree that we do not have sufficient evidence to claim that there is a general depletion of self-epitopes, and we have changed the text accordingly.

Splice isoforms lead to depletion of candidate epitopes: The authors make a big claim around the fact that splice isoforms can lead to depletion of other “candidate epitopes”, however, their methodology and claims are unclear. Are the “candidate epitopes” all epitopes (wildtype +mutated)? If so, this claim

is not surprising, since it is comparing only non-cancer epitopes from splice isoforms to any epitopes in the native isoform. A more interesting comparison would be to compare splice isoform derived neoepitopes to somatic mutation-derived neoepitopes in the same sample - to what extent to splice isoforms subsume somatic mutation-derived neoepitopes?

For our analyses we considered two types of epitopes. One type corresponds to tumor-specific splicing-derived neoepitopes. These are peptides with affinity to the MHC-I complex that are not encoded in the wild-type transcripts but are encoded in the altered ORF as a consequence of the tumor-specific splicing alteration. The second type corresponds to splicing-affected self-epitopes. These are peptides with affinity to the MHC-I complex that are encoded in the wild-type transcripts but would be not encoded (potentially depleted) in the altered ORF as a consequence of the tumor-specific splicing alteration. This is now clarified in the Methods section.

To measure the relation with mutation-related epitopes, we first calculated the overlap between events and mutations. As most of the splicing changes that we have analysed occur within introns far away from annotated exons, we used whole-genome sequencing data, which was only available for the SCLC samples (George et al. 2015). From all the events identified in SCLC samples that were changing the ORF, only a 6,16% of them overlapped with a somatic mutation (using the genomic extent of the event and 200nt around it).

We also calculated the mutation-derived neoepitopes using pvacseq (https://pubmed.ncbi.nlm.nih.gov/31907209/). This tool uses the same tools as ISOTOPE to identify candidate epitopes (NetHMC and NetHMCpan) and also uses the same affinity threshold (500nM). In general, we observed more splicing derived neoepitopes than mutation-associated (using RNA-seq and mutation data from the Pfeiffer et al. 2012 and George et al. 2015 cohorts):

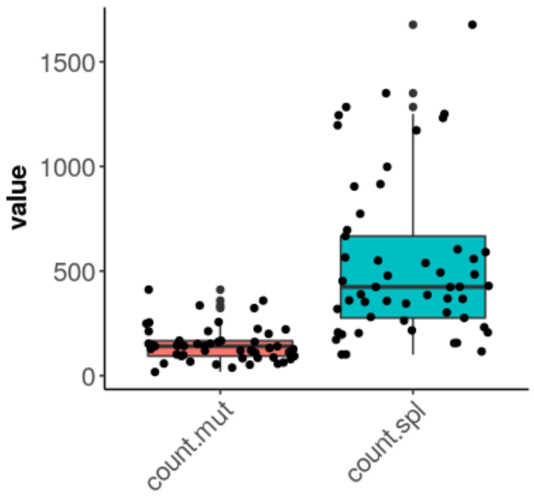

Additionally, when we looked at the relation between the number of splicing-derived and mutation-derived neoepitopes in each sample, we observed a weak correlation:

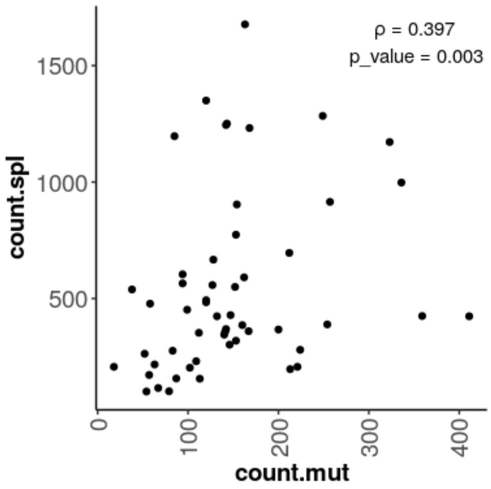

We also compared whether there were any overlapping candidate epitopes from both sets. Although the identification of the candidate epitopes was performed in both using the same method in exactly the same conditions (NetMHC and NetMHCPan, amino acid length from 8 to 11, epitopes with lower binding affinity than 500nm), there were no overlaps between candidates generated by somatic mutations and candidates generated by aberrant splicing changes. This indicates that aberrant splicing changes could provide a new repertoire of target neoantigens to the immunopeptidome, completely independent from that generated by somatic alterations.

We have added these results to the Supp. Fig. 5.

Figure 5: This figure, with multiple panels, is only referenced in passing. It is standard to require a reference to each panel in a figure for it to be included, and as is I do not believe this figure adds much to this manuscript as written. The authors should either eliminate this figure or include more writing describing the results in it.

In the revised version we have expanded the text related to this figure.

Analysis of data from immune checkpoint blockade treated patients.

Panels 6a and b: In each panel, the authors test a range of hypotheses and identify a single statistically significant one. The approach is problematic in that typically, a multiple hypothesis correction would be required. Furthermore, it appears that the proportion of epitopes lost in both healthy and control for the PD1/IR group is less than in CTLA4, even though these samples are both at pretreatment. So I would hypothesize there is potentially a batch effect present somewhere in this analysis. The authors should perform a more careful analysis, controlling for multiple hypothesis testing as well as potentially correcting batch effects in their data to ensure this result is correct.

Each comparison is a single test and it involves a different set of genes. That is, the same gene is not used in more than one comparison. The comparisons are performed using Wilcoxon tests between two distributions of the proportion of epitopes obtained from a specific splicing alteration, which occur in different genes. So there are 4 comparisons in total, one for each splicing event type, where the same patients (albeit different genes) are considered. We could use these 4 tests to perform multiple-test corrections. However, this will not change the fact that the comparisons are not significant.

Additionally, we are not comparing between different cohorts, but within each cohort. All the conclusions are drawn from comparisons within each study. There are some differences between the two patient datasets that might explain the differences observed in the number of events in Figure 5a. One of them is the tumor purity (see our Supp. Figure 4). Another one is the number of reads per sample. The anti-PD1 datasets have more reads (~66M reads per sample) and have longer reads (100nt) than the anti-CTL4 dataset (~45M per sample, 75nt). We also found more events in the anti-CTL4 cohort compared to the anti-PD1 cohort.

We looked at various properties in each of these patient cohorts to test for possible confounding effects with the therapy response. These plots are now included in the Supp. Figs 11 and 12.

The number of mapped reads in each clinical group does not vary significantly within each cohort:

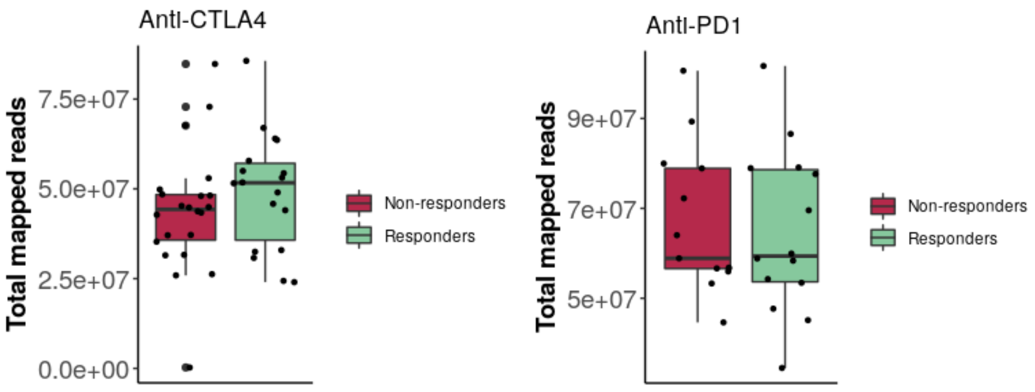

Moreover, except for a non-responder patient in the anti-CTLA4 group, the distribution of gene expression values (represented as log2(TPM)) is similar across the two clinical groups within each cohort:

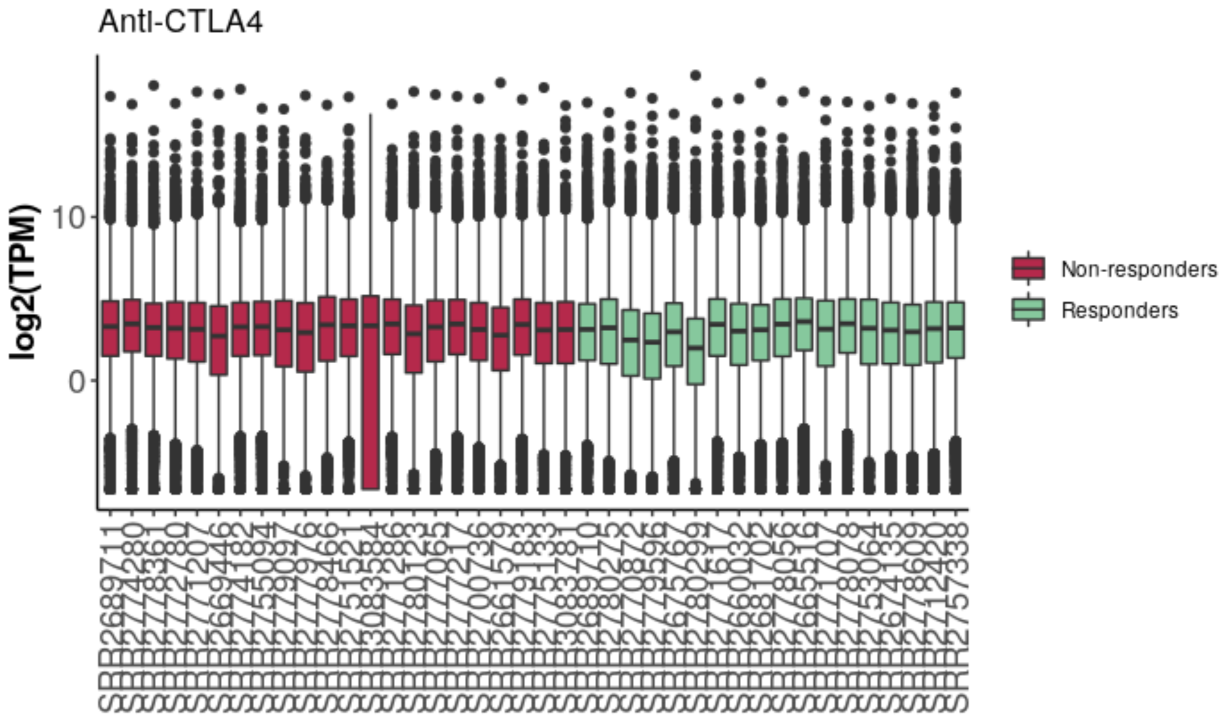

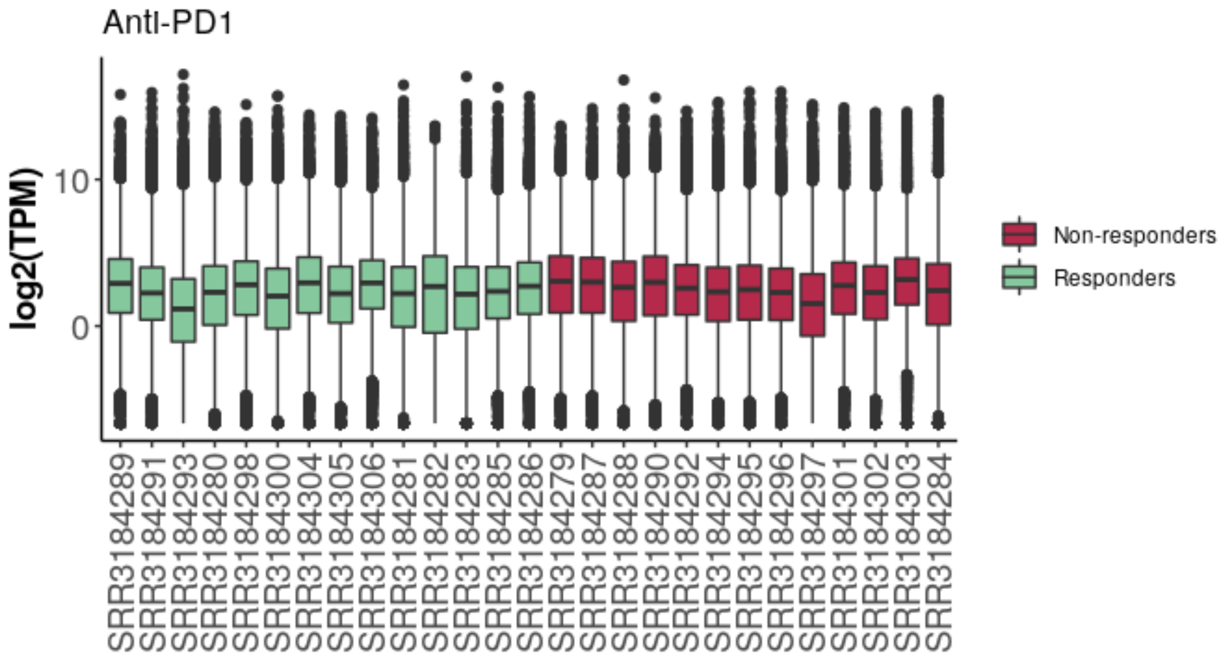

We also used ESTIMATE (Yoshihara et al, 2013) to measures immune infiltration, stromal content, and overall tumor purity in relation to the treatment response, We only detected some significant differences (p<0.05) in relation to the stromal content in the anti-PD1 cohort:

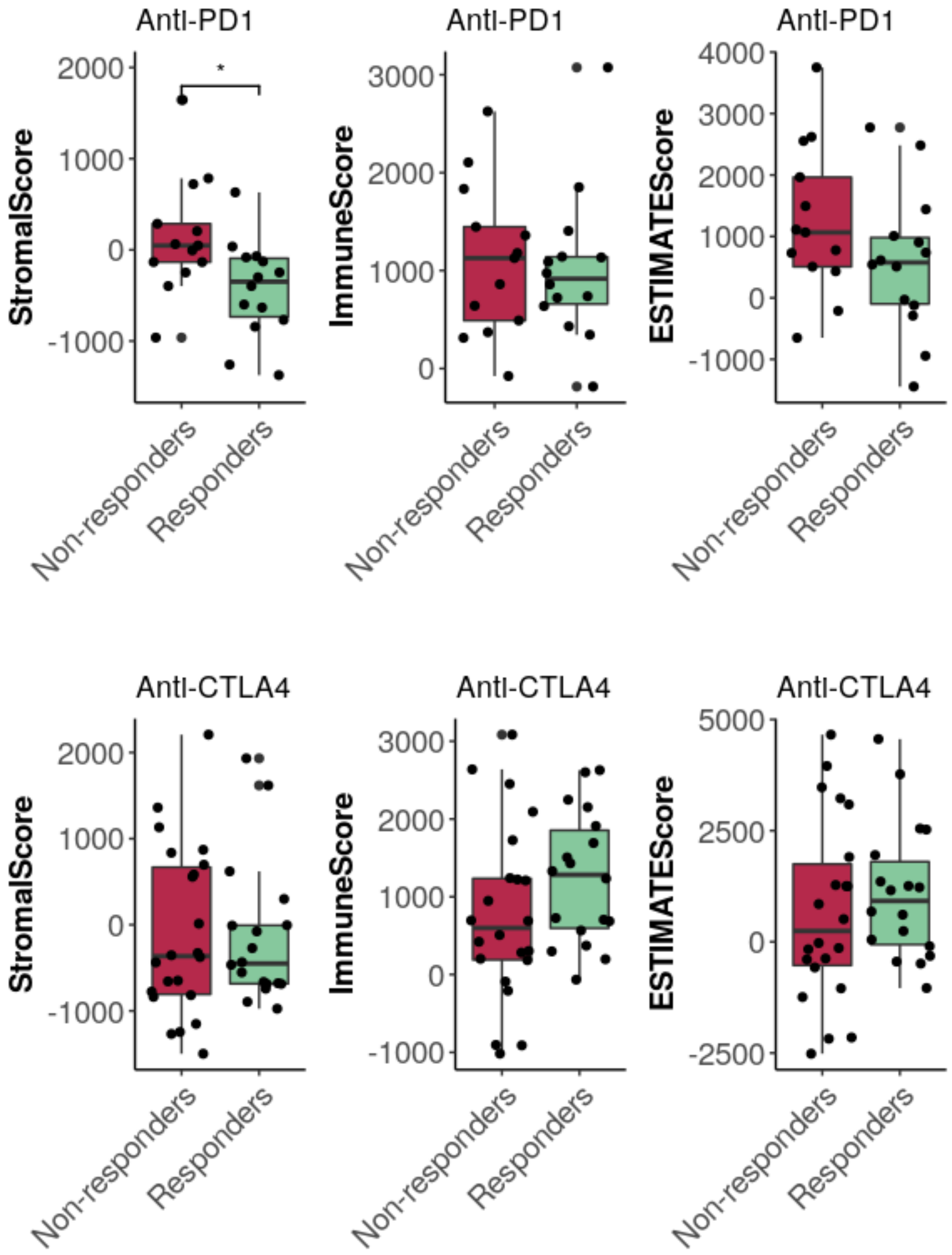

Stroma content can affect gene expression and splicing (see e.g. https://pubmed.ncbi.nlm.nih.gov/24335142/ and https://pubmed.ncbi.nlm.nih.gov/25578962/), so we

cannot discard that for the anti-PD1 there may be an effect on splicing due to the stroma content. On the other hand, there was no correlation between the number of splicing-neoepitopes and the estimated stromal content.

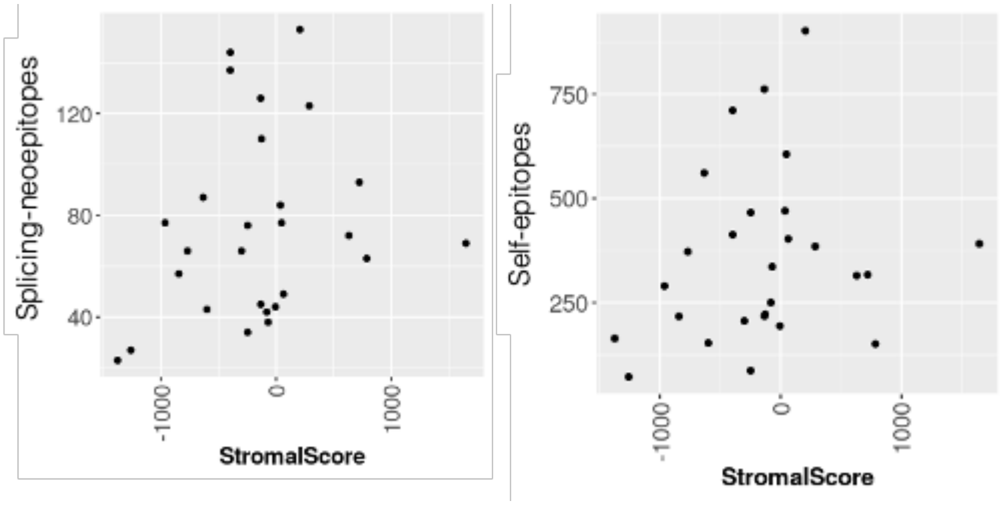

We have added these plots to Suppl. Figures 11 and 12.

Panels 6c, d, e: In general I find this analysis unconvincing, since it counts the clinical data from each patient multiple times (comparing R vs. NR, but among all epitopes, i.e, N=total number of epitopes, whereas the R vs. NR is among total number of patients). It is important in any analysis comparing clinical variables that each patient be counted only once (i.e., that N=the total number of patients). The authors should fix the issues with this analysis and perform an analysis with the appropriate comparator groups.

In each of these plots, we are comparing two distributions of epitopes, one from the responders (R) group, and one from the non-responders (NR) group. We do not know which are the relevant epitopes setting apart these two groups. Similarly, we also do not know whether the relevant epitopes are the same in all patients. In fact, no epitopes occur in all patients in a cohort (see Fig. 6f and Fig. 6g). As a consequence, we cannot test them individually across the clinical subgroups (responder / non-responder). This other comparison would require a multiple-test correction. On the other hand, we performed a comparison where we are testing the distribution of affinities of all epitopes produced in the R and NR groups. Our interpretation of these results is that the number of events and epitopes produced in the R and NR groups are not different, but they appear to have differences in binding affinity, and tend to appear in a low number of patients.

Reviewer #2:

1. Software and manual. I have downloaded the ISOTOPE. However, the authors wrote that "the scripts are ready to be run in a slurm cluster.", and I do not have access to one. It will be good to provide the version of the pipeline for stand-alone linux computers. Also, in the manual the authors

wrote that "Until all jobs generated by a part are finished do not run the following part.". It would be useful to provide a sequential script for this purposes. Overall, the manual should be more user-friendly and detailed.

Some steps are computationally intensive, especially the epitope evaluation, which is a third party software. This step and the bootstrapping test (empirical test to identify significant events) are thus parallelized. Therefore, it is not recommended to run the pipeline on a single computer, as it would not be efficient.

We have now provided a detailed description of the pipeline in GitHub: https://github.com/comprna/ISOTOPE with the detailed commands for all the analysis steps and a sample dataset to be used for testing purposes.

2. Statistical tests. The authors write that "ISOTOPE performs a bootstrapping test to establish the significance of the read support of the candidate splicing alterations. This type of test has not been performed in previous similar analyses of splicing-derived neoepitopes and ensures the robustness of the events detected". It is important to explain the choice of bootstrapping method and why bootstrapping is better than other statistical methods to assess the accuracy of estimates.

The objective was to assess the probability of observing a similar splicing alteration with similar or greater read support in the same gene locus in the same patient. The test worked as follows. For each tumor specific splicing, we selected 100 random regions from the same gene locus of the same size of the event to be tested. We then compared the read support for the event interest with the read support of the random events. Given that splicing changes are obtained by the mapped reads, with this test we can account for possible mapping biases and expression levels that may affect differently each gene locus. This empirical test also takes into account the possible differences in mapped reads between genes. We have changed “bootstraping” for “empirical” to make it more clear and have tried to improve the description of this method in the manuscript.

3. Structure of the manuscript. Some of the paragraphs in the results section may be more appropriate for the Methods. For example, discussion of the selection of method between PHLAT (Bai et al. 2014) and Seq2HLA (Boegel et al. 2018) may be more suitable for the Methods, or a Supplement.

This is an important result to validate the prediction of the HLA-type directly from the RNA-seq of a tumor sample, and has not been thoroughly tested before in similar analyses. We decided to test two of the most commonly used tools to predict the HLA-type from RNA-seq. This figure provides a validation of PHLAT to work on individual tumor samples, which is why we decided to include it in the ISOTOPE pipeline. Accordingly, we considered it would be important to maintain this in the results section. It is also standard to include this type of benchmarking analyses in the results section in articles describing methodologies.

4. Comparison with other available methods and tools has not been done.

We have not been able to identify a similar tool that is publicly available to make these comparisons. For instance, this publication https://pubmed.ncbi.nlm.nih.gov/30078747/ described a similar analysis using novel junctions connecting known exons with data from TCGA and validating the new peptides with standard mass-spec data. However, they did not provide a pipeline to run their analyses on

different datasets. Similarly, this publication https://pubmed.ncbi.nlm.nih.gov/30114007/ performed analysis of intron-retention (IR) events only in cell lines and validated the candidate epitopes with MHC-associated mass-spec data. However, the authors did not provide an open source pipeline that could be re-used in theirs or other datasets. There is a recent conference abstract describing a pipeline to identify neoepitopes generated by alternative splicing in B-cell lymphoblastic leukaemia https://ashpublications.org/blood/article/134/Supplement_1/1342/427222/Pipeline-for-Discovering-Neoepitopes-Generated-By but we have not been able to find a software repository for this pipeline.

In summary, there is no publication or preprint that provides a pipeline to calculate the tumor-specific splicing changes in a tumor sample and obtain candidate neoepitopes. Regarding the validation of the prediction of epitopes, all analyses previously published use the same methods, NetHMC and NetMHCpan, to evaluate the affinity to the MHC complex.

We have provided the complete description of the pipeline in the ISOTOPE GitHub (https://github.com/comprna/ISOTOPE). Our pipeline considers neoskipping events, IR events, as well as novel alternative splice-sites and de novo exonizations, therefore extending the spectrum of splicing variations from previous methods. We also extended the normal controls beyond GTEX to use junctions from the normal samples in Intropolis (splicing junctions measured from SRA samples), from the CHESS 2.0 annotation, and from matched lung normal samples from one of the analysed cohorts.

Reviewer #3: Trincado et al

ISOTOPE: ISOform-guided prediction of epiTOPES in cancer

This paper describes the creation of a novel bioinformatic pipeline to predict the change in MHC class I epitope profile in a tumor specimen using whole transcriptome RNAseq data. The pipeline is developed using cell line data, and its functionality is replicated on other cell lines and on tumor samples. Most interestingly, the ISOTOPE method predicts that many more self-epitopes are lost in tumor samples than neoepitopes are gained. The authors hypothesize that the loss of self-epitopes may be an important driver of immunogenicity in cancer, and that self-epitope loss may be a biomarker for response to immunotherapy.

While the observation that self-epitope loss is common in tumors is a provocative and interesting finding, it is not well-established what biological or clinical relevance this may have from this study. Analysis of a melanoma cohort is provided that does show that for one class of splicing alteration in one therapy type there is a small difference in proportion of immunotherapy responders and non-responders. However, it is hard to judge the significance of this finding in the absence of more clinical data or a better understanding of the underlying biology.

We appreciate the supporting comments. We have toned down the mentioned claims and have improved the description of the pipeline. We have left the implications of the identified epitopes for the discussion section.

Major Comments:

1. This paper would be strengthened significantly by better tying the phenomenon of self-epitope loss to a clinical or biological consequence. As the authors observe, the melanoma cohort used here is likely not ideal for analysis of this phenomenon, since mutation burden is very high in this tumor and the effects of epitope gain/loss due to splicing are likely diluted by mutation-generated neoepitopes. Analysis in another tumor type might be more fruitful. Admittedly, it is very difficult to find datasets like this to test the pipeline on, but showing a clear significance of the phenomenon would make it a lot more convincing.

We have applied the ISOTOPE pipeline to two independent cell line datasets with their corresponding MHC-associated mass-spec data (Rozanov et al, Ritz et al), to several SCLC patient cohorts (George et al., Rudin et al., Peifer et al,, Iwakawa et al.), and to two independent melanoma cohorts with annotation of their response to immunotherapy treatment

We would also like to clarify that we do not claim to have shown that the splicing alterations have a clinical significance. The main aim of our analyses was to show that splicing alterations of any kind could also contribute to the immunopeptidome and to provide a pipeline for that. We have analysed various tumor cohorts to test whether in those patients, the tumor-specific splicing-derived epitopes could play any role in relation to the characteristics of the tumor or to the response to immunotherapy.

Our results indicate that the splicing alterations contribute minimally to the tumor immunopeptidome in the cases analysed. We are limited by the type of data available for these and other tumors to be able to identify cases in which splicing alterations may play a more relevant role. On the other hand, we provide a pipeline and a detailed tutorial (https://github.com/comprna/ISOTOPE) to perform these analyses in other samples.

2. This study does take some steps to look at the relationship between Tumor Mutation Burden (TMB) and epitope gain/loss. Because TMB is the best understood measure of immunogenicity, it would be really helpful to get a sense of how TMB and epitope gain/loss are related. It is stated that for the SCLC dataset, TMB and the number of epitope gains/losses is not correlated based on Supplemental Figure 2. This does appear to be true, but a more rigorous treatment would be helpful, as this is an important point – what is numerically, the relationship between TMB and neoepitope gain, epitope loss, total epitope changes, etc.

We have followed the reviewer's suggestion and have calculated the correlations between the number of splicing alterations detected and the tumor mutation burden (TMB). We observed no correlation across all the events types (Spearman $\rho = 0.182$). When testing separately each event type, we observed again a low general correlation. However, there was a statistically significant correlation for Neoskipping events ($\rho = 0.42$):

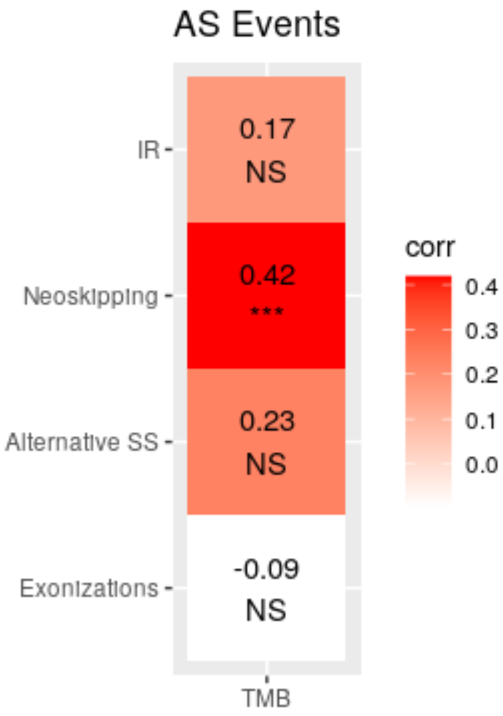

We did the same with the splicing-derived neoepitopes and self-epitopes. We observed again low correlation across all the event types between the TMB and the neo-epitopes (ρ = 0.194) and self-epitopes (ρ = 0.196). When looking independently at each event type, we observed a significant correlation only for Neoskipping events:

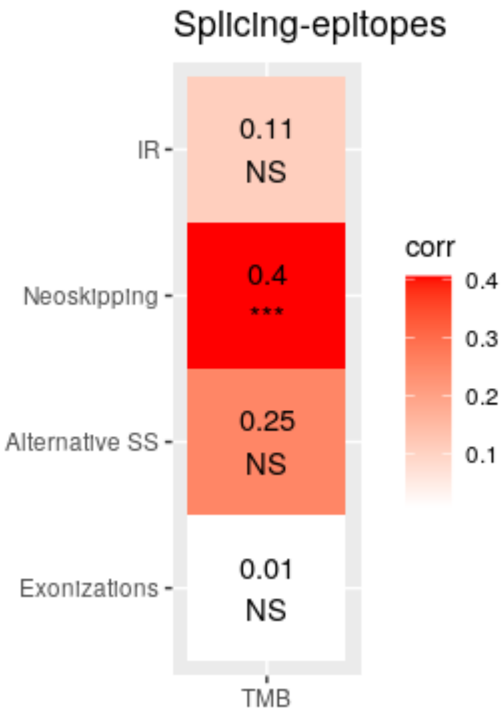

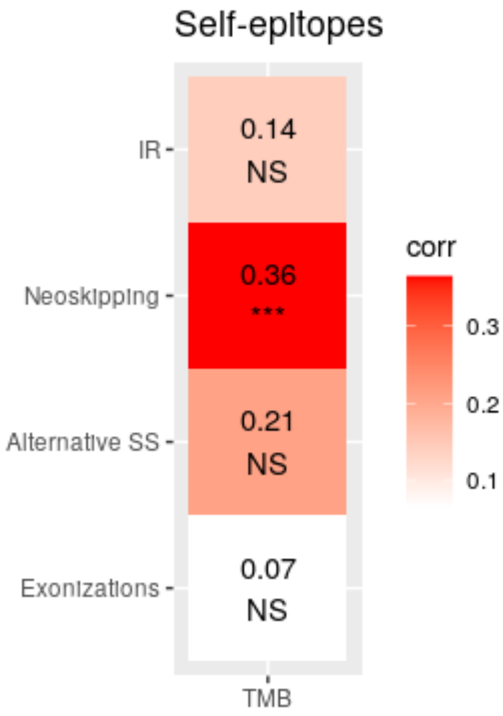

Further, there is a clear difference between the three cohorts used in this analysis – for epitope gains/losses, clearly Georg>Rudin>Iwakawa. Do the correlations/lack of correlations hold up within each cohort?

Testing each cohort independently, there are fewer events that the correlations are low in general. For George et al:

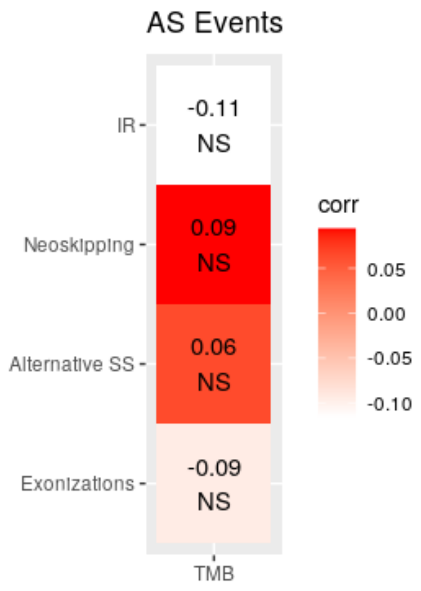

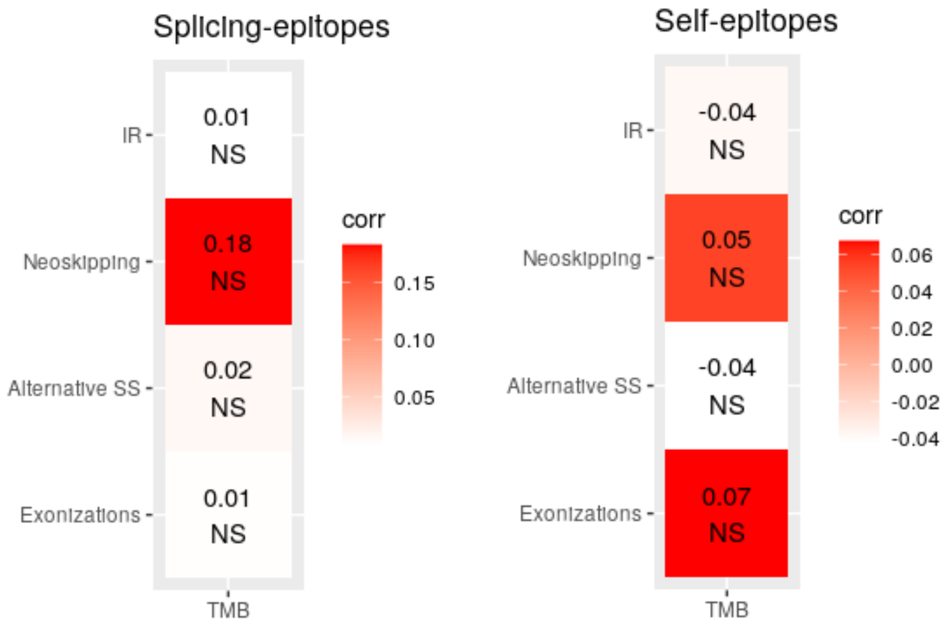

For Rudin et al:

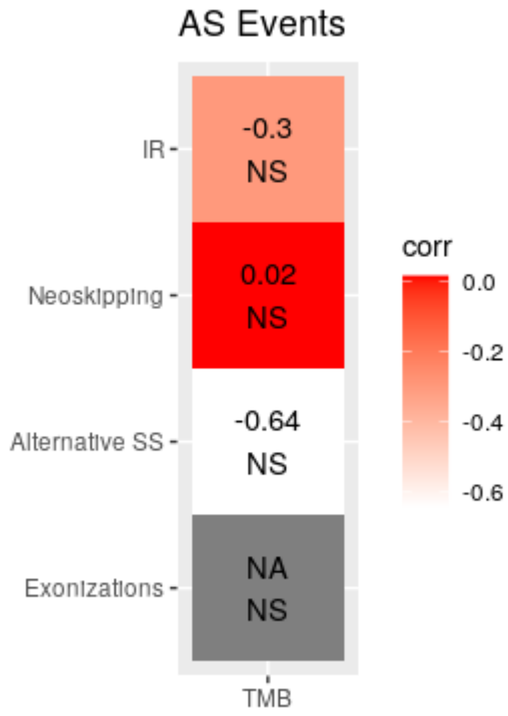

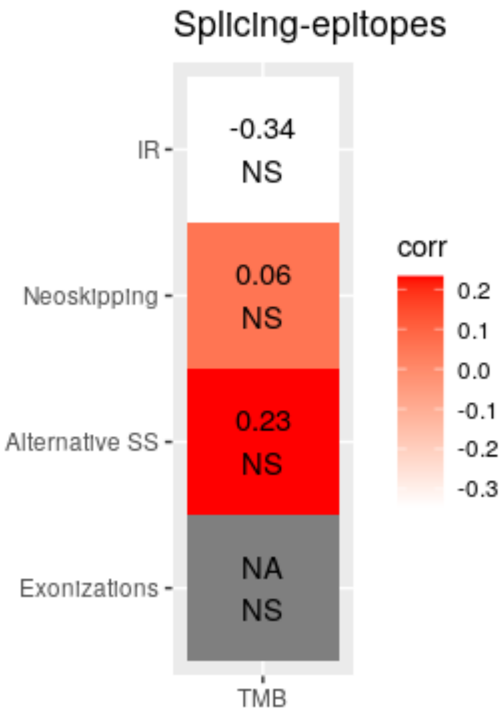

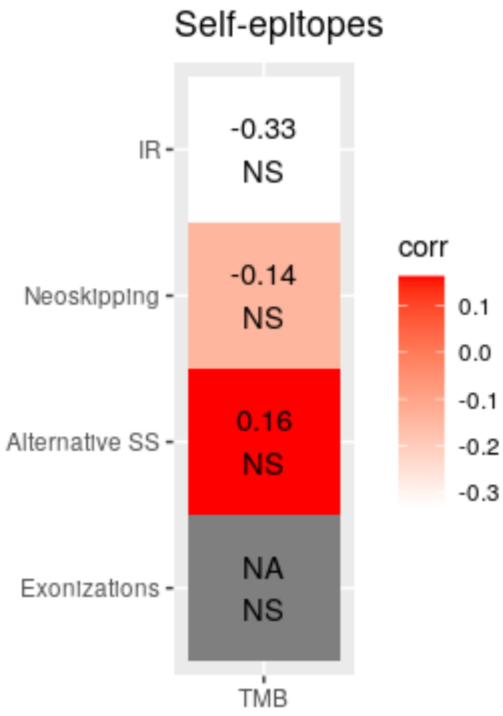

Iwakawa et al:

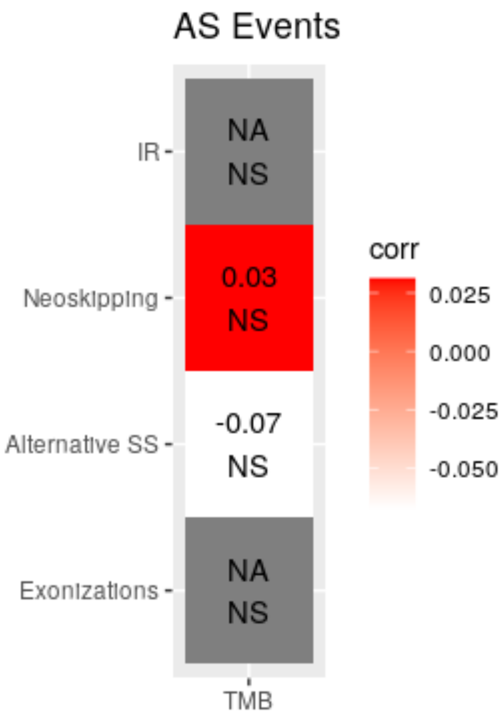

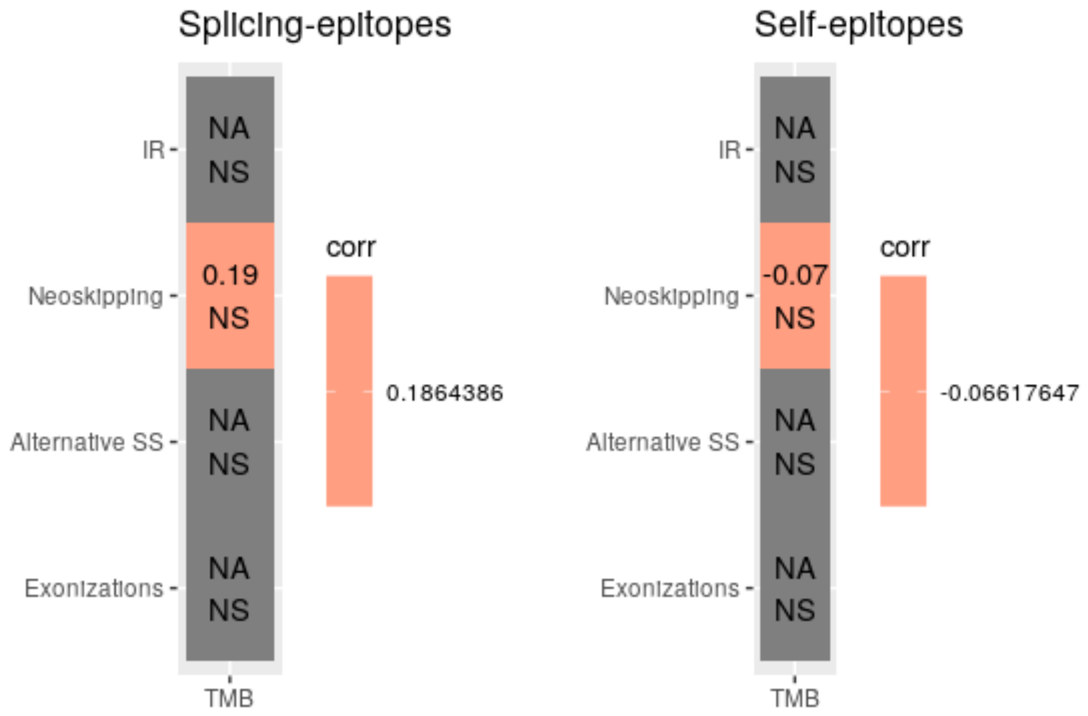

NS: non-significant.

NA: There are too few events / epitopes (<2) and the correlation cannot be estimated.

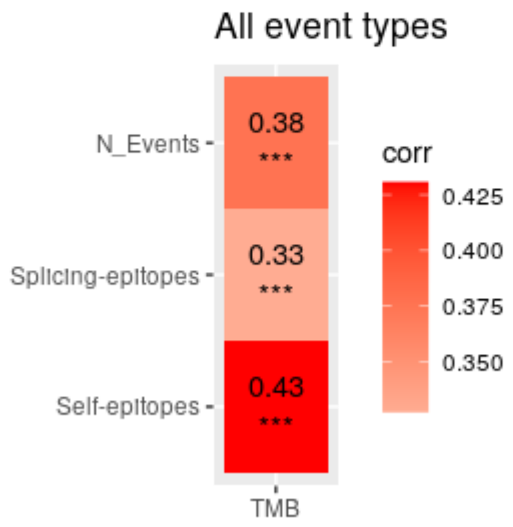

This analysis is now mentioned in the manuscript and the relevant plots are shown in Supp. Fig. 4.

I find it surprising that number of epitopes lost/gained does NOT correlate to TMB, since you'd think the same underlying process would be causative for both (i.e. DNA mutations). This is exciting, because a mutational signature that is not correlated to TMB may reflect some different underlying biology that independently affects immunogenicity, and which may be a powerful biomarker in combination with TMB.

Thank you for this positive comment. We agree that this would be an exciting possibility.

3. Analysis of all datasets is broken down by splicing mutation type. It would be helpful to see the overall data for each dataset with all mutation types lumped together, if there is no biological reason to think that a neoepitope or an epitope loss generated by each type of splicing event is different. The simplest model to test is that the overall level of epitope loss correlates to a clinical outcome, and it would be helpful to try to develop a single metric for this that could be used as a biomarker like TMB.

We agree with the reviewer that there should not be a biological difference between epitopes generated from different types of events. However, the number of intron retention events is generally higher than the rest of event types. As a result, when pooling all results together, the effects on the other types gets masked. When we pooled all event types together, i.e. pooling together the results within each cohort in Fig. 6a or Fig. 6b, we did not see any differences in the number of tumor-specific splicing-derived epitopes or splicing-affected self-epitopes in relation to the response to immunotherapy (see new Supp. Fig. 6a). We also pooled together the affinity data corresponding to figures 6c, 6d, and 6e, and we found no relation between affinity and response to therapy. We understand this is due to the large number of candidates produced that do not contribute to the difference. It is only when a subset is considered that the differences are apparent.

Minor comments:

-In figure 5A the colors for total peptides and peptides changed seem to have been reversed?

Thanks for pointing this out. We have changed the colors accordingly.

-Does the number of self-antigens lost correlate to the quantity of coding sequence lost? I can see how skipping an exon can remove an antigen, but it is less obvious how epitopes are lost in other mechanisms that add sequence (like intron retention). Are these all losses of epitopes that span splice junctions? Is it a result of sequence loss that accompanies gains?

An insertion of an intron generally introduces an in-frame stop coding, and the open reading frame (ORF) is shortened. So IR events can lead to shorter peptides. The self-epitopes affected by this are encoded downstream of the retained intron.

To provide a more general assessment of the lengths of the altered ORFs as a consequence of the tumor-specific splicing alterations, we have plotted the lengths of the WT transcript and the aberrant transcript produced.

Below we plot the ratio of these lengths in log2 scale, i.e. log2(WT length / aberrant length). The plots are separated according to whether the change involves the creation of a splicing-derived neoepitope only (blue), the removal of a splicing-affected self-epitope only (green), or both (red). We plot in the lower panels the proportion of the total corresponding to each case:

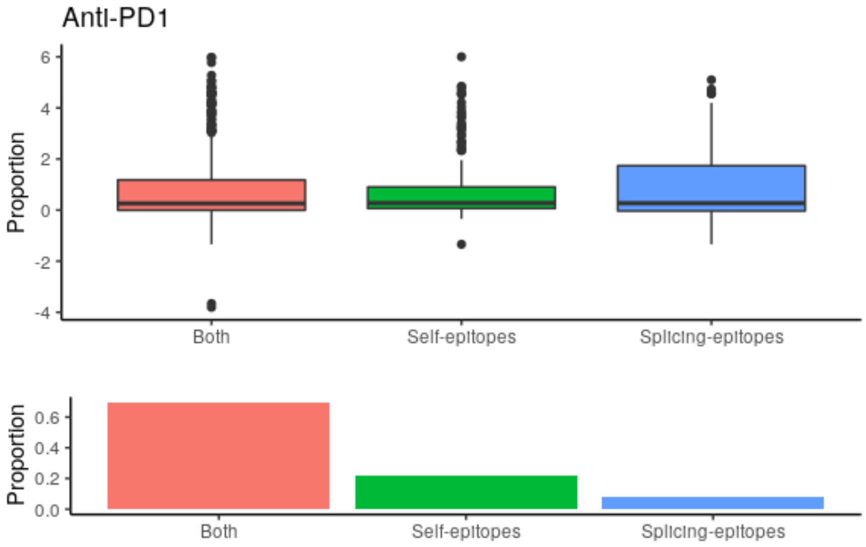

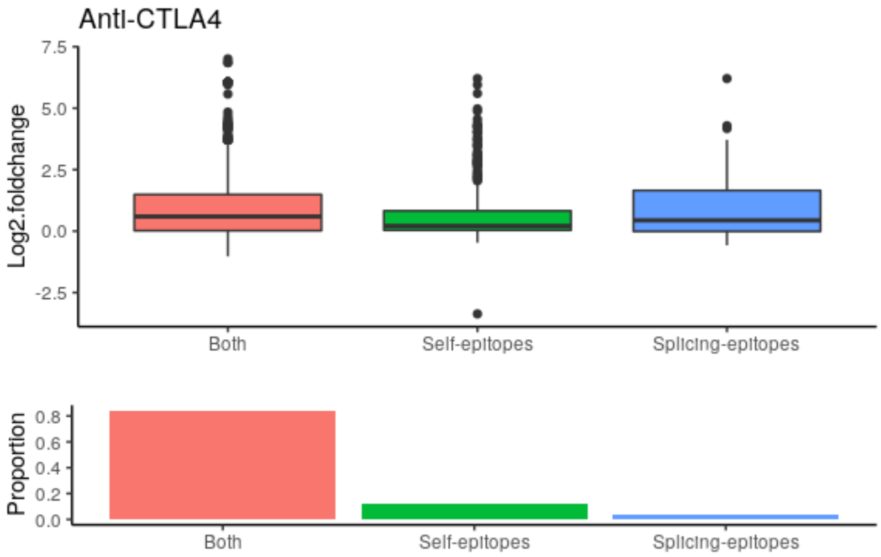

The distributions are centered above 0, hence the lengths of the aberrant transcripts are in general shorter than the WT. We also did not detect any significant difference between the events giving rise to new epitopes and the ones depleting epitopes only. In the majority of transcripts, the splicing change would be creating neo-epitopes and removing self-epitopes (red in the plots below). These cases also showed a tendency for the tumor isoform to be shorter than the normal isoform. This is in agreement with previous reports showing that tumor-specific isoforms are generally shorter than normal isoforms, e.g., https://pubmed.ncbi.nlm.nih.gov/28854369/.

These analyses are now mentioned in the manuscript and the plots are included in Supp. Fig. 6.

-Can the authors comment on the relative importance of the tumor/normal comparison and the use of splicing data from outside non-tumor datasets to define the set of “normal” splice junctions? Is it possible to devise a pipeline that doesn’t need a comparison to a normal patient sample for tumor-only sequencing, which is common in the clinical setting?

ISOTOPE can work for tumor-only analysis, since it predicts HLA-type from the same RNA-seq data from the tumor sample and compares against a large pre-established compendium of normal junctions. We used a large dataset of normal samples (GTEX, Intropolis normal samples, normal lung samples, CHESS annotation). With this we wanted to be comprehensive and eliminate any spliced junction that appeared in a broad range of normal samples. The importance of this set is that, as it includes normal tissues and cells from many individuals, it provides a comprehensive definition of "normal".

Our pipeline is already devised to work with a single tumor sample:

1) It calculates all splicing junctions in the sample
2) It filters those junctions that coincide with a set of normal junctions (derived from the comprehensive set mentioned above), which is provided by the ISOTOPE pipeline
3) It calculates the HLA type directly from the same tumor sample (hence our paragraph about the benchmarking of Phlat and Seq2HLA
4) It calculates the potential splicing-derived epitopes

Additionally, if MHC-associated mass-spec is available, the ISOTOPE pipeline can be used to validate those epitopes (commands included in ISOTOPE's GitHub https://github.com/comprna/ISOTOPE).

Thus, in the clinical setting, ISOTOPE could be used with an individual tumor sample.

To investigate novel prognostic signatures, one could apply ISOTOPE like we did to the melanoma cohorts.

How to work with ISOTOPE is now explained in detail with an example dataset in the GitHub: https://github.com/comprna/ISOTOPE

-In figure 6b the proportions of epitopes lost are much less for both responders and non-responders are much less (~75% vs ~95%) for intron retentions than for all other event categories shown and for IR in the CTLA4-treated group. Can the authors comment on why this may be? Also, is this significant to why this was the only segment of the data that showed a difference between responders and non-responders?

The anti-CTLA4 and anti-PD1 are two independent cohorts, sequenced by different labs. Thus it is expected that there may be different sources of variation between those two cohorts. IR events are also the most abundant splicing changes (Fig. 5a), and hence there will be a greater chance to identify IR events and IR-derived epitopes that separate the groups.

To further address the reviewer's comment, we looked into various properties of the responders and non-responder patients that could account for the observed difference, see new Supp. Figs. 11 and 12. We found no significant differences in the number of sequencing reads or in the expression distributions between clinical subgroups (shown now in Supp. Fig. 11). We also studied (shown now in Supp. Fig. 12) the differences between responders and non-responders in terms of stromal content, immune infiltration, and overall composition score using ESTIMATE (Yoshihara et al, 2013). There was a significant difference in relation to the stromal content in the anti-PD1 cohort:

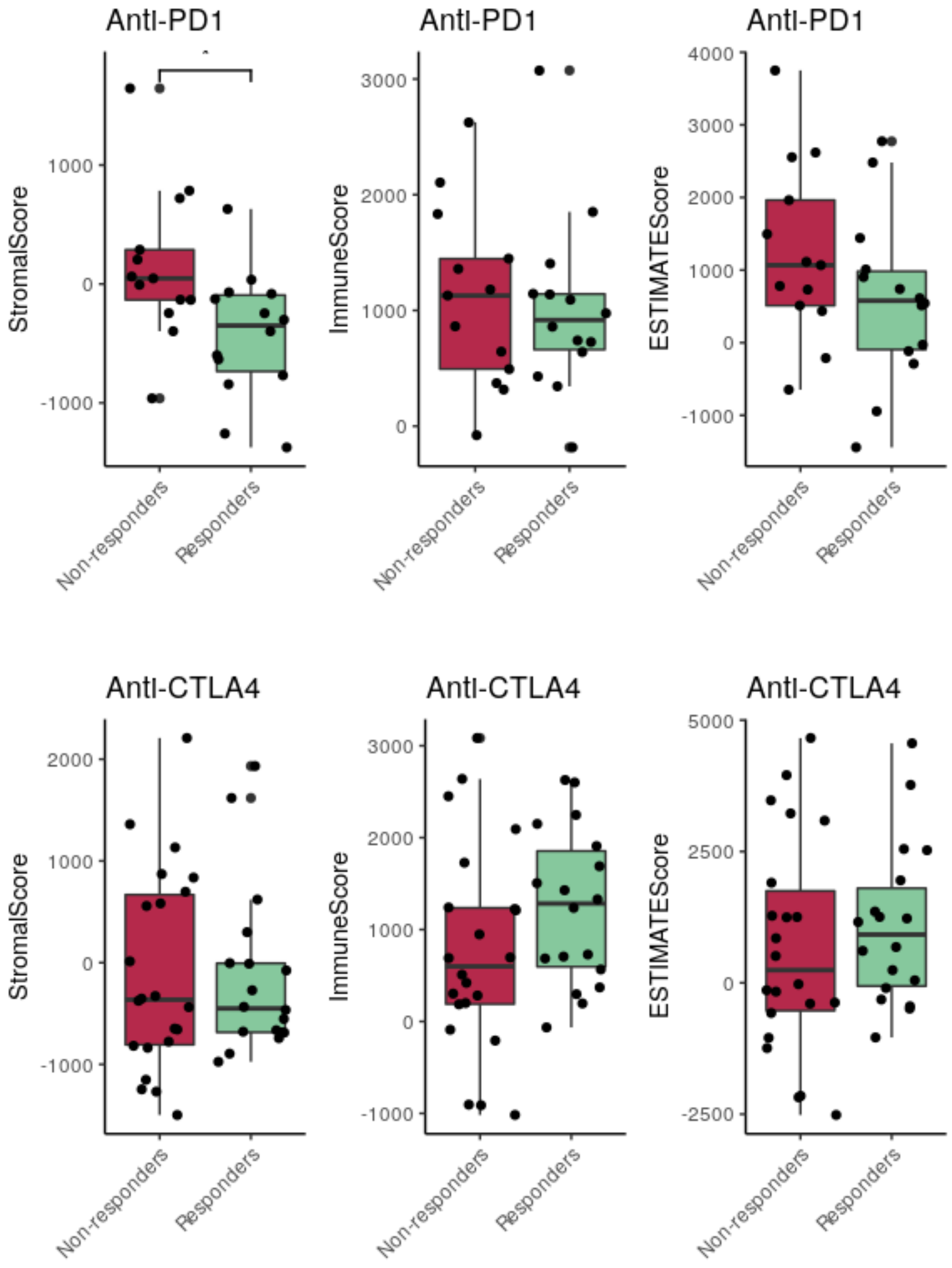

Stroma content can affect gene expression and splicing (see e.g. https://pubmed.ncbi.nlm.nih.gov/24335142/ and https://pubmed.ncbi.nlm.nih.gov/25578962/), so we

cannot discard that for the anti-PD1 cohort, part of the differences observed in the IR events are explained by the differences in stroma content. On the other hand, there was no correlation between the number of splicing-neoepitopes and the estimated stromal content:

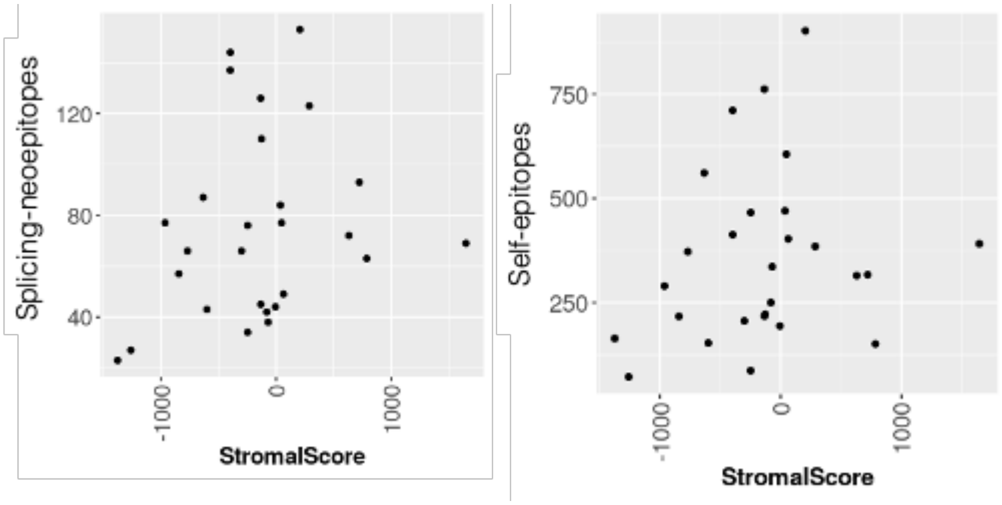

We now describe this result in the manuscript and have added these plots to Suppl. Figures 11 and 12.

- In support of Figure 6 it is stated that the number of splicing-generated neoepitopes is not different between responders and non-responders. I don't think these data are shown, so this should be at least cited as "data not shown", though I think it's an interesting analysis that should be included.

We show these plots below. There are no significant differences between responders and non-responders. We have added a supplementary figure with these distributions (Supp Fig 8a).

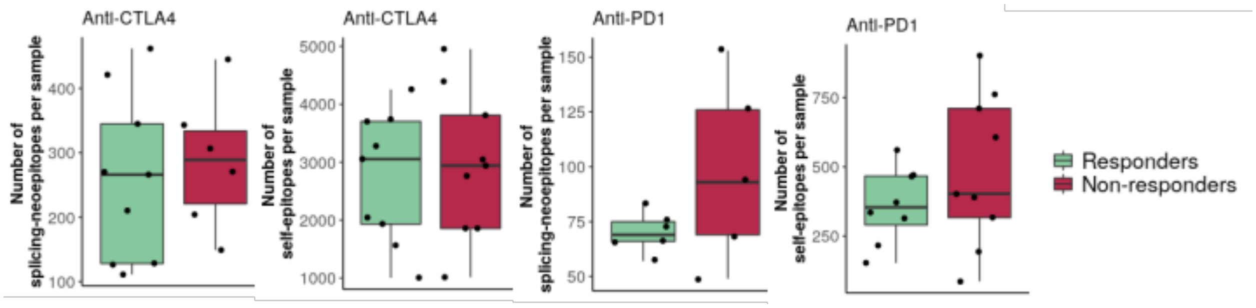

- Please make data labels bigger. I had a really hard time reading some of them. Also "A5_A3" isn't intuitive at all and should be replaced with "alternative splice site" as in figure 1

We have made labels bigger. We have also changed A5_A3 to "Alternative SS".

Have all data underlying the figures and results presented in the manuscript been provided?

Large-scale datasets should be made available via a public repository as described in the *PLOS Computational Biology* data availability policy, and numerical data that underlies graphs or summary statistics should be provided in spreadsheet form as supporting information.

Reviewer #1: Yes

Reviewer #2: Yes

Reviewer #3: None

All the data used in this article is already available from public repositories.

All the data corresponding to the analysis and the graphs are provided in the supplementary tables.
