# Supplementary material for: ISOTOPE: ISOform-guided prediction of epiTOPEs in cancer
Source: PLoS Comput Biol. 2021 Sep 16;17(9):e1009411. doi: 10.1371/journal.pcbi.1009411 (PMC8478223; doi:10.1371/journal.pcbi.1009411)
Supplement: Attachment — Submitted filename: answer_to_reviewers_v24.pdf [file pcbi.1009411.s021.pdf]

Answer to Editor's and Reviewer's comments in blue

Comments from reviewer 2 need to be fully addressed. In particular, version of the pipeline for stand-alone Linux computers to run on small test dataset need to be provided, this is important for providing convenient way for reviewers and readers to test and experiment with pipeline on small data sets.

We have re-implemented ISOTOPE so that it can be run locally on a single computer as well. We have provided the details in the same github page (https://github.com/comprna/ISOTOPE). Now, to run ISOTOPE in a computer cluster it is enough to use a flag in the command line (--cluster). Without that flag, ISOTOPE will run in the local computer. We have also added a toy dataset to test run the commands.

Supplemental tables value is currently limited by absence of gene symbols, this prevents readers from quickly and conveniently searching for isoform(s) in gene of interest.

We have added the gene symbols to the supplementary tables.

Reviewer #2: The manual remains to be not user-friendly. The hyperlinks were the test dataset did not work. Without the sample dataset it is not possible to evaluate the pipeline. Good user-friendly program needs to have a built-in dataset(s) and several well-described usage examples.

We have corrected the hyperlinks. We have also added a toy dataset and several examples. We have also added a version of ISOTOPE that can run on a single computer.

Regarding the comparison software, Part I has been implemented by many other tools (https://journals.plos.org/plosone/article?id=10.1371/journal.pone.0156132, https://bioconductor.org/packages/release/bioc/vignettes/SGSeq/inst/doc/SGSeq.html, https://arxiv.org/pdf/1405.0788.pdf). It is important to assess the quality of exon/intron predictions.

We agree that the comparison with other tools may help assess the quality of the predicted events. We showed in the past that this approach can be useful to identify high-confident alternative splicing events (https://pubmed.ncbi.nlm.nih.gov/29571299/). We also found that 1) different methods tend to have a low overlap of predictions, and that 2) events predicted by one method and not the others can still be validated experimentally.

With this caveat in mind, we have compared ISOTOPE results with LeafCutter (https://pubmed.ncbi.nlm.nih.gov/29229983/) for the small cell lung cancer (SCLC) samples from George et al. 2015. We measured whether the junctions that support the events generated by ISOTOPE had also support fromLeafCutter cluster:

**Event_type**	**Unique events produced by ISOTOPE**	**Supported also by LeafCutter**	**Proportion**
Exonizations	582	547	93,99

Alternative splice site	16694	10966	65,69
Neoskipping	4323	4281	99,03

Thus, 65%-99% of events predicted by ISOTOPE in the SCLC samples have also support from LeafCutter. The lower proportion of coincidences for alternative splice-sites could be due of the higher variability of in these sites (see e.g. https://pubmed.ncbi.nlm.nih.gov/21151575/), which result in variable predictions by different methodologies.

We could not compare IR events, since LeafCutter does not predict IR events.

LeafCutter was run using default parameters:
Minimum anchor length = 8,
Maximum intron length = 500000bp,
Minimum intron length = 50,
minimum fraction of reads in a cluster that support a junction = 0.001,
minimum reads in a cluster = 5,

Reviewer #3: My thanks to the authors for their thorough response to the reviewers' comments. My review is concerned mainly with the clinical utility and biological insight gained from this new method of analysis.

My major comment was that the study should attempt to better explain how this novel method for analysis of splicing alterations in tumors could be used clinically or provide biological insight. While the authors have not convincingly demonstrated an immediate use for their method, I think they have done what's possible with public data, and that further investigation is probably a new study and outside of the scope of this report. My comments have been appropriately answered on all other counts.

We would like to thank the reviewer for the encouraging comments and support.
We have added a text in the discussion that addresses the potential clinical utility and biological insights gained from our new analysis method.
